# Chemotherapy weakly contributes to predicted neoantigen expression in ovarian cancer

**DOI:** 10.1186/s12885-017-3825-0

**Published:** 2018-01-22

**Authors:** Timothy O’Donnell, Elizabeth L. Christie, Arun Ahuja, Jacqueline Buros, B. Arman Aksoy, David D. L. Bowtell, Alexandra Snyder, Jeff Hammerbacher

**Affiliations:** 1 Icahn School of Medicine at Mount Sinai, New York, NY USA; 2 Peter MacCallum Cancer Centre, East Melbourne, Victoria, 3002 Australia; 3 Department of Medicine, Memorial Sloan-Kettering Cancer Center, New York, NY USA; 4 Adaptive Biotechnologies, Seattle, WA USA; 5 Department of Microbiology and Immunology, Medical University of South Carolina, Charleston, SC USA

**Keywords:** Neoantigen, Mutational signature, Chemotherapy

## Abstract

**Background:**

Patients with highly mutated tumors, such as melanoma or smoking-related lung cancer, have higher rates of response to immune checkpoint blockade therapy, perhaps due to increased neoantigen expression. Many chemotherapies including platinum compounds are known to be mutagenic, but the impact of standard treatment protocols on mutational burden and resulting neoantigen expression in most human cancers is unknown.

**Methods:**

We sought to quantify the effect of chemotherapy treatment on computationally predicted neoantigen expression for high grade serous ovarian carcinoma patients enrolled in the Australian Ovarian Cancer Study. In this series, 35 of 114 samples were collected after exposure to chemotherapy; 14 are matched with an untreated sample from the same patient. Our approach integrates whole genome and RNA sequencing of bulk tumor samples with class I MHC binding prediction and mutational signatures extracted from studies of chemotherapy-exposed *Caenorhabditis elegans* and *Gallus gallus* cells. We additionally investigated the relationship between neoantigens, tumor infiltrating immune cells estimated from RNA-seq with CIBERSORT, and patient survival.

**Results:**

Greater neoantigen burden and CD8+ T cell infiltration in primary, pre-treatment samples were independently associated with improved survival. Relapse samples collected after chemotherapy harbored a median of 78% more expressed neoantigens than untreated primary samples, a figure that combines the effects of chemotherapy and other processes operative during relapse. The contribution from chemotherapy-associated signatures was small, accounting for a mean of 5% (range 0–16) of the expressed neoantigen burden in relapse samples. In both treated and untreated samples, most neoantigens were attributed to COSMIC *Signature (3)*, associated with BRCA disruption, *Signature (1)*, associated with a slow mutagenic process active in healthy tissue, and *Signature (8)*, of unknown etiology.

**Conclusion:**

Relapsed ovarian cancers harbor more predicted neoantigens than primary tumors, but the increase is due to pre-existing mutational processes, not mutagenesis from chemotherapy.

**Electronic supplementary material:**

The online version of this article (doi:10.1186/s12885-017-3825-0) contains supplementary material, which is available to authorized users.

## Background

Many chemotherapies including platinum compounds [1], cyclophosphamide [2], and etoposide [3] exert their effect through DNA damage, and recent studies have found evidence for chemotherapy-induced mutations in post-treatment acute myeloid leukaemia [4], glioma [5], and esophageal adenocarcinoma [6]. Successful development of immune checkpoint-mediated therapy [7] has focused attention on the importance of T cell responses to somatic mutations in coding genes that generate neoantigens [8]. Patients with more CD8+ T cell infiltration in their tumors have better prognosis [9, 10], and studies based on bulk-sequencing of tumor samples followed by computational peptide-class I MHC affinity prediction [11] have associated increased mutations and resulting mutant MHC I peptide ligands with improved survival [12], especially in the context of checkpoint blockade immunotherapy [13, 14]. Ovarian cancers fall into an intermediate group of solid tumors in terms of mutational load present in pre-treatment surgical samples [15]. However, the effect of standard chemotherapy regimes on tumor mutation burden and resulting neoantigen expression in ovarian cancer is poorly understood.

Investigators associated with the Australian Ovarian Cancer Study (AOCS) performed whole genome and RNA sequencing of 79 pre-treatment and 35 post-treatment cancer samples from 92 high grade serous ovarian carcinoma (HGSC) patients, including 12 patients with both pre- and post-treatment samples [16]. The samples were obtained from solid tissue resections, autopsies, and ascites drained to relieve abdominal distension. Treatment regimes varied but primary treatment always included platinum-based chemotherapy. In their analysis, Patch et al. reported that post-treatment samples harbored more somatic mutations than pre-treatment samples and exhibited evidence of chemotherapy-associated mutations. Here we extend these results by quantifying the mutations and predicted neoantigens attributable to chemotherapy-associated mutational signatures. We find that, while neoantigen expression increases after treatment and relapse, only a small part of the increase is due to mutations associated with chemotherapy signatures. To assess the relevance of neoantigens in HGSC, we also interrogate associations between predicted neoantigen burden, immune cell infiltration estimated from RNA-seq, and patient survival, and find that neoantigen burden and CD8+ T cell infiltration are non-redundant survival predictors in this cohort.

## Methods

### Clinical sample information

We grouped the AOCS samples into three sets — “primary/untreated,” “primary/treated,” and “relapse/treated” — according to collection time point and prior chemotherapy exposure (Table 1). The primary/untreated group consists of 75 primary debulking surgical samples and 4 samples of drained ascites. The primary/treated group consists of 5 primary debulking surgical samples obtained from patients pretreated with chemotherapy prior to surgery (neoadjuvant chemotherapy). The relapse/treated group consists of 24 relapse or recurrence ascites samples, 5 metastatic samples obtained in autopsies of two patients, and 1 solid tissue relapse surgical sample, all of which were obtained after prior exposure to one or more lines of chemotherapy. In summary, these groupings yield 79 primary/untreated samples, 5 primary/treated samples, and 30 relapse/treated samples. Specimen and clinical information for each sample is listed in Additional file 1.

**Table 1:** Sample counts by tissue and prior chemotherapy exposure

	Patients	Samples (with an untreated sample from same patient)				
		Solid tissue	Ascites	Total		
Primary/untreated	76	75	4	79		
Primary/treated	5	5 (0)	0 (0)	5 (0)		
Relapse/treated	23	6 (4)	24 (10)	30 (14)		
Total	92	86 (4)	28 (10)	114 (14)		
	*Carboplatin*	*Cisplatin*	*Cyc.*	*Etoposide*	*Gemcitabine*	*Paclitaxel*
Primary/treated	5 (0)	0 (0)	0 (0)	0 (0)	1 (0)	4 (0)
Relapse/treated	30 (14)	5 (2)	10 (6)	1 (1)	17 (8)	30 (14)
Total	35 (14)	5 (2)	10 (6)	1 (1)	18 (8)	34 (14)

Parentheses indicate chemotherapy-treated samples with a patient-matched primary/untreated sample. Cyc., cyclophosphamide

Independent of treatment, ascites samples trend toward harboring more detected mutations, perhaps due to increased intermixing of clones. We therefore stratified by tissue type (solid tumor or ascites) when comparing the mutation and neoantigen burdens of pre- and post-treatment samples. As some patients provided multiple samples of the same type, we reweighted the samples so each patient contributes equally in these comparisons.

### Mutation calls

We analyzed the mutation calls published by Patch et al. [16] (Additional file 2). DNA and RNA sequencing reads were downloaded from the European Genome-phenome Archive under accession EGAD00001000877. Adjacent single nucleotide variants (SNVs) from the same patient were combined to form multinucleotide variants (MNVs).

We considered a mutation to be present in a sample if it was called for the patient and more than 5% of the overlapping reads and at least 6 reads total supported the alternate allele. We considered a mutation to be expressed if there were 3 or more RNA reads supporting the alternate allele. In the analysis of paired pre- and post-treatment samples from the same donors, we defined a mutation as unique to the post-treatment sample if the pre-treatment sample contained greater than 30 reads coverage and no variant reads at the site.

### Variant annotation, HLA typing, and MHC binding prediction

Protein coding effects were predicted using Varcode (manuscript in preparation, https://github.com/openvax/varcode). All transcripts overlapping each mutation were considered, and the transcript with the most disruptive effect was selected using a prioritization similar to other tools (from highest priority: frameshift, loss of stop codon, insertion or deletion, substitution). In the case of frameshift mutations, all downstream peptides generated up to a stop codon were considered potential neoantigens.

HLA typing was performed using a consensus of seq2HLA [17] and OptiType [18] across the samples for each patient (Additional file 3).

Class I MHC binding predictions were performed for peptides of length 8–11 using NetMHCpan 2.8 [19] with default arguments (predicted neoantigens are listed in Additional file 2).

### Mutational signatures

The use of mutational signatures is necessary because it is not possible to distinguish chemotherapy-induced mutations from temporal effects when comparing primary and relapse samples by mutation count alone. A mutational signature ascribes a probability to each of the 96 possible single-nucleotide variants, where a variant is defined by its reference base pair, alternate base pair, and base pairs immediately adjacent to the mutation. Signatures have been associated with exposure to particular mutagens, age related DNA changes, and disruption of DNA damage repair pathways due to somatic mutations or germline risk variants in melanoma, breast, lung and other cancers [20], and provide a means of identifying the contribution that chemotherapy may make to the mutations seen in post-treatment samples. For example, the chemotherapy temozolomide has been shown to induce mutations consisting predominantly of *C*→*T* (equivalently, *G*→*A*) transitions at CpC and CpT dinucleotides [5]. To perform deconvolution, the SNVs observed in a sample are tabulated by trinucleotide context, and a combination of signatures, each corresponding to a mutagenic process, is found that best explains the observed counts. Mutational signatures may be discovered *de novo* from large cancer sequencing projects but for smaller studies it is preferable to deconvolve using known signatures [21].

The Catalogue Of Somatic Mutations In Cancer (COSMIC) Signature Resource curates 30 signatures discovered in a pan-cancer analysis of untreated primary tissue samples. While signatures for exposure to the carboplatin/paclitaxel combination that is standard first line therapy in ovarian cancer have not been established, two recent reports provide data on mutations detected in cisplatin-exposed *Caenorhabditis elegans* [22] and a *Gallus gallus* (chicken) cell line exposed to several chemotherapies including cisplatin, chyclophosphamide, and etoposide [23]. As cisplatin is thought to induce the same DNA adducts as carboplatin, we reasoned that the mutational signatures of these related compounds are likely similar [24]. In the AOCS cohort, 28 patients with post-treatment samples were treated with carboplatin, four with cisplatin, eight with cyclophosphamide, and one with etoposide.

From the SNVs identified in the animal models, we defined two signatures for cisplatin, a signature for cyclophosphamide, and a signature for etoposide (Additional file 4: Figures S1 and S2). As both studies sequenced replicates of chemotherapy-treated and untreated (control) samples, identifying a mutational signature associated with treatment required splitting the mutations observed in the treated group into background and treatment effects. We did this using a Bayesian model for each study and chemotherapy drug separately.

Let *C*_*i*,*j*_ be the number of mutations observed in experiment *i* for mutational trinucletoide context 0≤*j*<96. Let *t*_*i*_∈{0,1} be 1 if the treatment was administered in experiment *i* and 0 if it was a control. We estimate the number of mutations in each context arising due to background (non-treatment) processes *B*_*j*_ and the number due to treatment *T*_*j*_ according to the model:

$$C_{i,j} \sim \mathit{Poisson}(B_{j} + t_{i} T_{j}) $$

We fit this model using Stan [25] with a uniform (improper) prior on the entries of *B* and *T*. The treatment-associated mutational signature *N* was calculated from a point estimate of *T* as:

$$N_{j} = \left (\frac{T_{j}}{\sum_{j'}{T_{j'}}} \right) \left (\frac{h_{j}}{m_{j}} \right) $$

where *h*_*j*_ and *m*_*j*_ are the number of times the reference trinucleotide *j* occurs in the human and preclinical model (*C. elegans* or *G. gallus*) genomes, respectively.

Signature deconvolution was performed with the deconstructSigs [21] package using the 30 mutational signatures curated by COSMIC [26] extended to include the putative chemotherapy-associated signatures (Additional files 5 and 6). When establishing whether a signature was “detected” in a sample, we applied the 6% of SNVs cutoff recommended by the authors of the deconstructSigs package. Signatures assigned weights less than this threshold in a sample were considered undetected.

To estimate the number of SNVs and neoantigens generated by a signature, for each mutation in the sample we calculated the posterior probability that the signature generated the mutation, as described below. The sum of these probabilities gives the expected number of SNVs attributable to each signature. For neoantigens, we weighted the terms of this sum by the number of neoantigens generated by each mutation.

Suppose a mutation occurs in context *j* and sample *i*. We calculate Pr[*s*∣*j*], the probability that signature *s* gave rise to this mutation, using Bayes’ rule:

$$\Pr[s \mid j] = \frac{\Pr[j \mid s] \Pr[s]}{\sum_{s'}{\Pr[j \mid s']\Pr[s']}} = \frac{H_{s,j} \, D_{i,s}}{\sum_{s'}{H_{s',j} \, D_{i,s'}}} $$

where *D*_*i*,*s*_ is the result matrix from deconstructSigs, giving the contribution of signature *s* to sample *i*, and *H*_*s*,*j*_ is the weight for signature *s* on mutational context *j*. For each chemotherapy-associated signature, *H*_*s*,*j*_ is given by *N*_*j*_ above. For the other signatures it is defined in the COSMIC Signature Resource.

For treated samples with a pre-treatment sample available from the same patient, we deconvolved signatures for both the full set of mutations and for the mutations detected only after treatment. When calculating Pr[*s*∣*j*] for these samples, for each mutation we selected the appropriate deconvolution matrix *D*_*i*,*s*_ depending on whether the mutation was unique to the post-treatment sample.

### Survival analysis

Survival analyses were performed with the Cox proportional hazards model from the lifelines Python package version 0.9.3.2 (https://github.com/CamDavidsonPilon/lifelines). Analyses were stratified by tumor stage (III or IV) and included the logarithm of the tumor cellularity percentage as a covariate. Key findings were reproduced with the survival R package (https://cran.r-project.org/web/packages/survival).

### Immune deconvolution

RNA-seq reads were aligned using STAR version 2.4.1d [27], transcript quantifications were obtained with Cufflinks version 2.2.1 [28], and immune deconvolution was performed by CIBERSORT version 1.01 [29].

## Results

### Cisplatin and cyclophosphamide mutational signatures correlate with clinical treatment

We identified mutational signatures for cisplatin, cyclophosphamide, and etoposide from the *G. gallus* cell line data (Additional file 4: Figure S1), as well as a second cisplatin signature from experiments in *C. elegans* (Additional file 4: Figure S2). The two cisplatin signatures were not identical. Both signatures placed most probability mass on *C*→*A* mutations, but differed in preference for the nucleotides adjacent to the mutation. The *G. gallus* signature was relatively indifferent to the 5’ base and favored a 3’ cytosine, whereas the *C. elegans* signature was specific for a 5’ cytosine and a 3’ pyrimidine. The *G. gallus* cisplatin signature was closest in cosine distance to COSMIC *Signature (24) Aflatoxin*, *Signature (4) Smoking*, and *Signature (29) Chewing tobacco*, all associated with guanine adducts. The *C. elegans* cisplatin signature was similar to *Signature (4) Smoking*, *Signature (20) Mismatch repair*, and *Signature (14) Unknown*. The *G. gallus* cyclophosphamide signature favored *T*→*A* and *C*→*T* mutations and was most similar to COSMIC Signatures *(25)*, *(8)*, and *(5)*, all of unknown etiology. The *G. gallus* etoposide signature distributed probability mass nearly uniformly across mutation contexts and was most similar to COSMIC *Signature (5) Unknown*, *Signature (3) BRCA*, and *Signature (16) Unknown*. Overall, the chemotherapy signatures were no closer to any COSMIC signatures than the two most similar COSMIC signatures (*Signature (12) Unknown* and *Signature (26) Mismatch repair*) are to each other, suggesting that deconvolution could in principle distinguish their contributions.

We performed signature deconvolution on each sample’s SNVs (top and middle of Additional file 4: Figures S3 and S4). Detection of the cyclophosphamide signature at the 6% threshold was associated with clinical cyclophosphamide treatment (Bonferroni-corrected Fischer’s exact test *p*=0.004), occurring in 4/10 samples taken after cyclophosphamide treatment, 2/79 pre-treatment samples, and 2/25 samples exposed to chemotherapies other than cyclophosphamide. In contrast, the two cisplatin signatures were found in no samples, and the etoposide signature was found only in four pre-treatment samples.

**Fig. 1:**
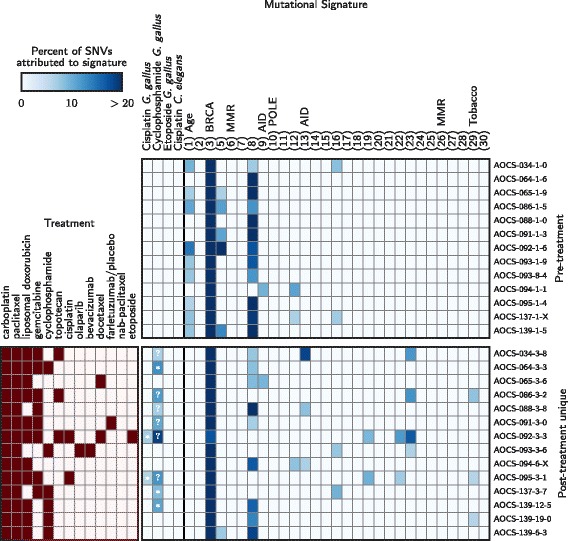
Detected mutational signatures for donor-matched primary/untreated and relapse/treated samples. *(Top)* Signatures detected in the pre-treatment samples. The first four signatures were extracted from reports of a *G. gallus* cell line and *C. elegans* after exposure to chemotherapy, and the rest are COSMIC curated signatures. COSMIC signature numbers are shown in parentheses, and the associated mutagenic process is indicated when known. Signatures not shown were undetected in these samples. *(Bottom)* Clinical treatments and detected signatures for the mutations unique to the post-treatment samples. Cases where a chemotherapy signature is detected are annotated with a (*) if the patient received the associated drug and a (?) otherwise

For better sensitivity, we next focused on the 14 relapse/treated samples from the 12 patients with both pre- and post-treatment samples. For each patient, we extracted the mutations that had evidence exclusively in the treated samples. Of 206,766 SNVs in the post-treatment samples for these patients, 93,986 (45%) satisfied our filter and were subjected to signature deconvolution (Fig. 1, bottom of Additional file 4: Figures S3 and S4). Within this subgroup, the *G. gallus* cisplatin signature was identified only in the two samples taken after cisplatin therapy, a significant association (*p*=0.04). The *C. elegans* cisplatin signature was detected in no samples, and the cyclophosphamide signature was detected in 3/6 cyclophosphamide-treated samples, but, unexpectedly, also in 6/8 non-cyclophosphamide-treated samples. These included the two post-treatment samples in which the signature was detected in the earlier analysis plus four additional samples. COSMIC *Signature (3) BRCA* and *Signature (8) Unknown etiology* were detected in 14/14 and 9/14 post-treatment samples, respectively, but *Signature (1) Age* was absent, consistent with its association with a slow mutagenic process operative before oncogenesis.

Considering all relapse/treated samples, the *G. gallus* cisplatin signature showed a dose-dependent relationship with the total number of cisplatin or carboplatin chemotherapy cycles administered (Pearson correlation *r*=0.47; Additional file 4: Figure S5). In a linear regression, each additional cycle of platinum was associated with 9.0 (95% CI 3.6–14.3) more genome-wide mutations attributed to this signature, giving rise to 0.08 (-0.01–0.17) additional neoantigens per cycle. A weaker trend was observed among patients whose only platinum exposure was carboplatin (*r*=0.24; 3.7 (-1.6–9.0) mutations per cycle). The cyclophosphamide signature had little association with the number of cycles of cyclophosphamide (*r*=0.11; 30.4 (-58.9–120.0) mutations per cycle). The time elapsed between the most recent chemotherapy cycle and sample collection did not independently correlate with total mutations or mutations attributed to chemotherapy signatures in a linear model that included the total number of cycles (*p*>0.15 for all tests).

In summary, the mutational signatures for cisplatin and cyclophosphamide extracted from experiments of a *G. gallus* cell line showed significant but inexact associations with clinical chemotherapy exposure.

### Pre-treatment neoantigens and CD8+ T cell infiltrate independently predict survival

Across the cohort, we identified 17,689 mutated peptides predicted to bind autologous MHC class I with affinity 500 nm or tighter [30]. All but 21 (0.12%) of these predicted neoantigens were private to a single patient (shared neoantigens are listed in Additional file 7).

Patients with greater predicted neoantigen burden in their solid tissue primary/untreated samples had longer survival (Additional file 4: Figure S6). In a Cox proportional hazards regression analysis, neoantigen count (hazard ratio (HR) of mean/variance standardized count: 0.73 (95% CI 0.54–0.97), *p*=0.03) and expressed neoantigen count (HR=0.75 (0.56–0.99), *p*=0.04) were significantly associated with survival, whereas mutation burden exhibited a weaker trend (HR=0.85 (0.65–1.11), *p*=0.2). This analysis excludes sample AOCS-166-1-2, from a patient with a germline deficiency in mismatch repair and by far the most mutations. If this sample is included in the analysis, the estimated effects of neoantigen count (HR=0.77 (0.57–1.02), *p*=0.07) and expressed neoantigen count (HR=0.78 (0.59–1.03), *p*=0.08) are relatively unaffected, but mutation burden is skewed (HR=1.0 (0.69–1.44), *p*=0.9).

Immune infiltrate deconvolution of each sample’s RNA-seq was performed using CIBERSORT, which estimates the relative abundance of 22 immune cell types as a fraction of the total immune infiltrate (Additional file 4: Figures S7 and S8). One sample (AOCS-056-1-X) failed deconvolution and was excluded from these analyses. Samples taken before or after chemotherapy treatment were broadly similar in immune infiltrate. Nonactivated macrophages (M0 macrophages) were the most prevalent population in both solid tissue (median 22% of infiltrate (range 0–54%)) and ascites (26% (3–57)) samples. Other myeloid lineage cells were also prevalent in ascites, including activated mast cells (9% (0–69)) and monocytes (6% (0–50)). Follicular helper (solid tissue: 10% (0–23); ascites: 5% (1–24)) and memory resting (9% (0–31); 6% (0–28)) CD4+ T cells were the most abundant lymphocyte populations in both solid tissue and ascites samples.

CD8+ T cells accounted for a median of 3% (0–30) of infiltrate in solid tissue samples and were uniformly rare in ascites (0% (0–3)). In primary/untreated solid tissue samples, greater CD8+ T cell infiltrate was associated with improved survival (HR=0.65 (0.47–0.88), *p*=0.006; Additional file 4: Figure S6). This appeared to be an independent effect from neoantigen burden. No significant correlation was observed between neoantigens and CD8+ T cell infiltrate, although among ascites samples increased total mutational burden was narrowly associated with increased CD8+ T cell infiltrate (*p*=0.03, Additional file 4: Figure S9). Both CD8+ T cell infiltrate and neoantigen burden were independently associated with survival in a Cox multiple regression analysis (CD8 T cells: HR=0.61 (0.44–0.85), *p*=0.003; neoantigens: HR=0.72 (0.54–0.97), *p*=0.03).

Among relapse/treated ascites samples, no significant survival association was observed for mutations (HR=1.2 (0.73–2.1), *p*=0.43), neoantigens (HR=0.81 (0.47–1.4), *p*=0.44), expressed neoantigens (HR=0.67 (0.387–1.15), *p*=0.14), or CD8+ T cell infiltrate (HR=1.2 (0.741–1.99), *p*=0.44). In a search over all other immune cell subsets, none showed significant survival association after correction for multiple hypothesis testing.

### Neoantigen burden increases at relapse

**Fig. 2:**
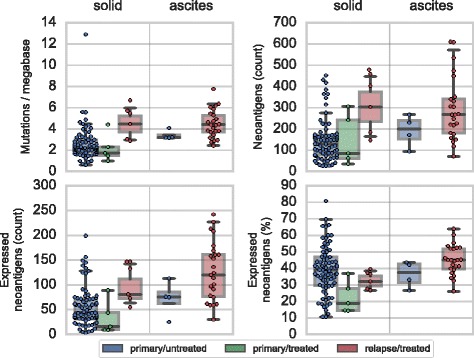
Comparison of mutation and neoantigen burden of chemotherapy-treated and untreated samples. Mutations (upper left), neoantigens (upper right), and expressed neoantigens by count (lower left) and as a percent of total neoantigens (lower right) are shown for primary/untreated samples (blue; solid tumor *n*=75, ascites *n*=4), primary/treated samples (green; solid tumor *n*=5), and relapse/treated samples (red; solid tumor *n*=6 samples from 3 patients, ascites *n*=24 samples from 21 patients). The shaded boxes indicate the interquartile region and the median line; multiple samples of the same type from the same patient have been reweighted so that each patient contributes equally. Points indicate individual samples

Relapse/treated samples harbored a median 78% more expressed neoantigens than primary/untreated samples (weighted mean of stratum-specific estimates). In particular, solid tissue relapse samples harbored a median of 71% (bootstrap 95% CI 23–123) more mutations, 107% (32–187) more neoantigens, and 72% (16–137) more expressed neoantigens than primary/untreated solid tissue samples (Fig. 2), all significant increases (Mann-Whitney *p*<0.05 for each of the three tests). A similar trend was observed for ascites samples. Relapse/treated ascites samples harbored 32% (14–51), 55% (10–118), and 83% (22–178) more mutations, neoantigens, and expressed neoantigens than primary/untreated ascites samples, respectively (*p*=0.07,0.10,0.05 for the three tests). This trend was also apparent in a comparison of paired samples from the same donors and in an analysis using a Bayesian modeling methodology (Additional file 4: Figure S10 and Additional file 8).

In contrast, primary/treated samples, which were exposed to neoadjuvant chemotherapy (NACT) prior to surgery, did not exhibit increased numbers of mutations, neoantigens, or expressed neoantigens, and in fact trended toward decreased neoantigen expression. The five primary/treated samples, all from solid tissue resections, harbored a median of 16 (9–89) expressed neoantigens compared to the median of 44 (39–60) observed in primary/untreated solid tissue samples, due to both fewer neoantigens in the DNA (median of 85 (36–306) vs. 130 (108–150)) and a lower rate of expression (median 19 (14–37) vs. 39 (36–42)% of neoantigens). This trend did not reach significance (Mann-Whitney *p*=0.08).

### Chemotherapy signatures weakly contribute to neoantigen burden at relapse

**Fig. 3:**
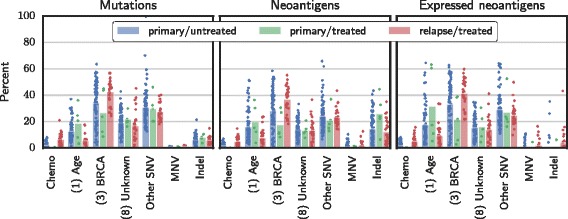
Contribution of key SNV signatures, MNVs, and indels on mutations *(left)*, neoantigens *(center)*, and expressed neoantigens *(right)*. The *Chemo* category combines the contributions from the chemotherapy signatures. COSMIC signature numbers are in parentheses. The *Other SNV* category represents SNVs not accounted for by the signatures shown. Bars give the mean, and points indicate individual samples

While we cannot determine with certainty whether any particular mutation was chemotherapy-induced, we can estimate the fraction of mutations and neoantigens attributable to each signature in a sample (Fig. 3 and Additional file 4: Figure S11).

Similarly to results reported by Patch et al., the most prevalent mutational signatures in this cohort were COSMIC *Signature (3)*, associated with BRCA disruption, *Signature (8)*, of unknown etiology, and *Signature (1)*, associated with spontaneous deamination of 5-methylcytosine, a slow process active in healthy tissue that correlates with age. These signatures together accounted for a median of 67% (95% CI 66–69) of mutations, 58% (56–61) of neoantigens, and 68% (67–71) expressed neoantigens across samples. These rates did not substantially differ with chemotherapy treatment.

The chemotherapy signatures accounted for a small but detectable part of the increased neoantigen burden of relapse samples. In primary/untreated samples, which indicate the background rate of chance attribution, chemotherapy mutational signatures accounted for a mean 2% of mutations (range 0–8), 2% (0–7) of the neoantigens, and 2% (0–8) of the expressed neoantigens. In each of the five primary/treated samples, less than 1% of the mutation, neoantigen, and expressed neoantigen burdens were attributed to chemotherapy signatures. For the relapse/treated samples, chemotherapy signatures accounted for a mean of 6% (range 0–21) of the mutations, 5% (0–15) of the neoantigens, and 5% (0–16) of the expressed neoantigens. The highest attribution to chemotherapy signatures occurred in sample AOCS-092-3-3, a relapse/treated sample from a patient who received two lines of carboplatin and three lines of cisplatin, the most in the cohort. For this sample, 21% (or approximately 3200 of 15,491) of the SNVs, 15% (9 of 61) of the neoantigens, and 16% (5 of 30) of the expressed neoantigens were attributed to chemotherapy signatures.

Signature deconvolution considers only SNVs, but studies of platinum-induced mutations have also reported increases in the rate of dinucleotide variants and indels. Indeed, we observed more MNVs overall and specifically the platinum-associated MNVs *CT*→*AC* and *CA*→*AC* reported by Meier et al. [22] in treated patients in both absolute count and as a fraction of mutational burden (*p*<10^−6^ for all tests). Sample AOCS-092-3-3, previously found to have the most chemotherapy-signature SNVs, also had the most platinum-associated dinucleotide variants and the second-most MNVs overall. This sample harbored 59 *CT*→*AC* or *CA*→*AC* mutations, compared to a mean of 3.2 (2.2–4.4) across all samples. Treated samples also harbored more indels in terms of absolute count (*p*=10^−4^). Overall, while MNVs and indels generate more neoantigens per mutation than SNVs, they are rare, comprising less than 3% of the mutational burden and 13% of the neantigens in every sample (Fig. 3), making it unlikely that chemotherapy-induced MNVs and indels have a large impact on neoantigen burden.

## Discussion

In this analysis of neoantigens predicted from DNA and RNA sequencing of ovarian cancer tumor and ascites samples, relapse samples obtained after chemotherapy exposure had a median of 78% more expressed neoantigens than untreated primary samples. However, putative chemotherapy mutational signatures accounted for no more than 16% of the expressed neoantigen burden in any sample. Most of the increase was instead attributable to mutagenic processes already at work in the primary samples, including COSMIC *Signature (3) BRCA* and *Signature (8) Unknown etiology*.

It is likely that many of the mutations considered unique to the relapse/treated samples were actually present before treatment, but were confined to too few cells to be detected. After surgery and adjuvant chemotherapy, outgrowth of a subclone would bring such private mutations to population levels detectable by bulk sequencing in the relapse/treated samples. Consistent with this interpretation, NACT-treated samples, which were exposed to chemotherapy as large tumors and for a short duration (typically 3 cycles), did not show increased mutation or neoantigen burden over untreated samples and had very few mutations attributed to chemotherapy.

Our results suggest it would be difficult to rationally increase neoantigen burden through chemotherapy, as even the most heavily treated patients show only a modest number chemotherapy-associated neoantigens. The patient with the most such neoantigens, AOCS-092, had 9/61 neoantigens attributed to chemotherapy. At the relapse/treated time point, no significant survival association for neoantigens was observed. In the Cox proportional hazards model fit to the primary/untreated samples, where neoantigens did correlate with survival, 9 additional neoantigens are predicted to improve two year survival rate by only 1.1% points (from 43.7% to 44.8%). These analyses are in the context of standard treatment regimes; neoantigens may have a greater impact for patients treated with immunotherapies. However, as immunotherapy trials in HGSC have focused on heavily pre-treated patients with recurrent disease, the substantially increased total neoantigen burden at recurrence is evidently not sufficient on its own for immunotherapy to be effective for many patients [31–34]. Other factors, perhaps unique ascitic or systemic immunosuppressive mechanisms, may also need to be overcome.

Remarkably, in primary/untreated samples, neoantigen burden was more closely associated with survival than overall mutation burden. This did not appear to be mediated by concomitant CD8+ T cell infiltration in the primary tumor; both factors independently associated with survival. Previous reports have found neoantigens and CD8+ T cell infiltrate to be favorable prognostic markers in a variety of tumor types and clinical contexts [9, 10, 12, 35, 36]. Analyses of both factors, however, have generally found a positive association between neoantigen burden and markers of tumor infiltrating lymphocytes [37, 38]. Our smaller sample size may explain the lack of significant association. Alternatively, some tumors with low neoantigen burden and high immune infiltration may have experienced selective loss of neoantigens, a process termed immunoediting [39]. One study reported a depletion of neoantigens, relative to what would be expected from the silent mutation rate, in several cancer types, but no significant effect in ovarian cancer samples with whole exome sequencing deposited in The Cancer Genome Atlas [37]. While we did not pursue it here, the present dataset would be an interesting cohort to re-evaluate such an effect.

The signatures for cisplatin and, to a lesser extent, cyclophosphamide extracted from the *G. gallus* experiments showed a modest correlation with clinical treatment, whereas the *G. gallus* etoposide and *C. elegans* cisplatin signatures were not detected in chemotherapy-exposed samples. The latter signature may be less accurate than the *G. gallus* cisplatin signature because it was derived from fewer mutations (784 vs. 2633). The number of mutations attributed to the *G. gallus* cisplatin signature correlated with the number of cycles of platinum chemotherapy; a weak trend held among patients treated only with carboplatin. In the case of cyclophosphamide, deconvolution of all mutations identified the signature in 4/10 samples treated with cyclophosphamide and 4/104 unexposed samples. However, when we focused on mutations detected uniquely in the post-treatment samples, 6/8 samples exposed only to non-cyclophosphamide chemotherapies exhibited the signature. As it was rarely detected in pre-treatment samples, this signature may reflect the effect of other (non-cyclophosphamide) chemotherapy.

A contrast to our results is a report of NACT temozlomide-treated glioma, in which it was reported that over 98% of mutations detectable with bulk sequencing in some samples were attributable to temozolomide [5]. Whether this difference is due to the drug used or disease biology requires further study.

We predicted a median of 64 (50–75) expressed MHC I neoantigens across all samples in the cohort, significantly more than the median of 6 reported by Martin et al. in this disease [40]. However, Martin et al. did not consider indels, MNVs, or multiple neoantigens that can result from the same missense mutation, used a 100 nm instead of 500 nm MHC I binding threshold, used predominantly lower quality (50 bp) sequencing, and only explicitly considered HLA-A alleles. Predicted neoantigen burden is best considered a relative measure of tumor foreignness, not an absolute quantity readily comparable across studies.

This study has several important limitations. Most critically, as the signatures may differ from actual effects in patients, chemotherapy-induced mutations could erroneously be attributed to non-chemotherapy signatures. This would result in an underestimation of the impact of chemotherapy. However, the fraction of mutations that do not match COSMIC signatures (1), (3), or (8) or a chemotherapy signature, a quantity indicated as “Other SNV” in Fig. 3, is no greater in the treated vs. untreated samples. This is evidence against a scenario in which many chemotherapy-induced mutations are unaccounted-for in our analysis because they do not match any signature or spuriously match other COSMIC signatures. However, we cannot exclude the possibility that chemotherapy-induced mutations could be erroneously attributed to COSMIC Signatures (1), (3), or (8). Experiments using human cell lines exposed to the range of chemotherapies used in recurrent ovarian cancer may fully address this question.

As this study is based on bulk DNA sequencing of heterogeneous clinical samples, the analysis is limited to mutations that are present in at least 5-10% of the cells in a sample. Data from Patch et al. suggests that even late-stage disease remains polyclonal, therefore potentially obscuring the impact of chemotherapy on the tumor genome. Single-cell sequencing may be required to observe most chemotherapy-induced mutations, especially in the neoadjuvant setting. However, while we may have been unable to detect highly subclonal mutations, it is expected that such clones would be unable to trigger an anti-tumor immune response that is effective against the bulk of the tumor [41].

Finally, this study does not consider neoantigens resulting from structural rearrangements such as gene fusions and relies on only 35 post-chemotherapy samples.

## Conclusion

In this study, we demonstrate a method for connecting mutational signatures extracted from studies of mutagen exposure in preclinical models with computationally predicted neoantigen burden in clinical samples. We found that relapsed HGSC tumors harbor a median of 78% more expressed neoantigens than untreated primary samples, and that cisplatin and cyclophophamide chemotherapy treatments account for a small but detectable part of this effect. The mutagenic processes responsible for most mutations at relapse are similar to those operative in primary tumors, with COSMIC *Signature (3) BRCA*, *Signature (1) Age*, and *Signature (8) Unknown etiology* accounting for most mutations and predicted neoantigens both before and after chemotherapy.

## Additional files

Additional file 1 Samples. Sample identifiers, basic clinical and chemotherapy information, specimen purities, mutation and neoantigen burden, contributions of major mutational signatures to mutations and neoantigens, and CIBERSORT immune infiltrate estimates. (CSV 224 kb)

Additional file 2 Mutations. Somatic variants and their read counts, predicted effects, and resulting neoantigens. (CSV 105000 kb)

Additional file 3 HLA types. Patient HLA types. (CSV 5 kb)

Additional file 4 Supplemental figures. **Figures S1–S11**. (PDF 798 kb)

Additional file 5 Mutational signatures. COSMIC signatures and extracted chemotherapy signatures. (CSV 5 kb)

Additional file 6 Signature deconvolutions. Results of mutational signature deconvolution, including a separate analysis of mutations unique to the treated paired samples. (CSV 42 kb)

Additional file 7 Shared neoantigens. Neoantigens predicted for multiple patients. (CSV 1 kb)

Additional file 8 Supplemental statistical notebook. Bayesian models to compare mutation, neoantigen, and expressed neoantigen burdens in treated and untreated samples. (PDF 980 kb)

## Abbreviations

AOCS: Australian ovarian cancer study
COSMIC: The catalogue of somatic mutations in cancer
HGSC: High grade serous ovarian carcinoma
indel: An insertion or deletion mutation
MNV: Multi nucleotide variant
NACT: Neoadjuvant chemotherapy
SNV: Single nucleotide variant
