# Supplementary material for: Chemotherapy weakly contributes to predicted neoantigen expression in ovarian cancer
Source: BMC Cancer. 2018 Jan 22;18:87. doi: 10.1186/s12885-017-3825-0 (PMC5778667; doi:10.1186/s12885-017-3825-0)
Supplement: Supplementary file 4 — Supplemental figures. Figures S1–S11. (PDF 798 kb) [file 12885_2017_3825_MOESM4_ESM.pdf]

Supplemental Figures

Chemotherapy weakly contributes to predicted neoantigen expression in ovarian cancer

Timothy O'Donnell, Elizabeth L. Christie, Arun Ahuja, Jacqueline Buros, B. Arman Aksoy, David D. L. Bowtell, Alexandra Snyder, Jeff Hammerbacher

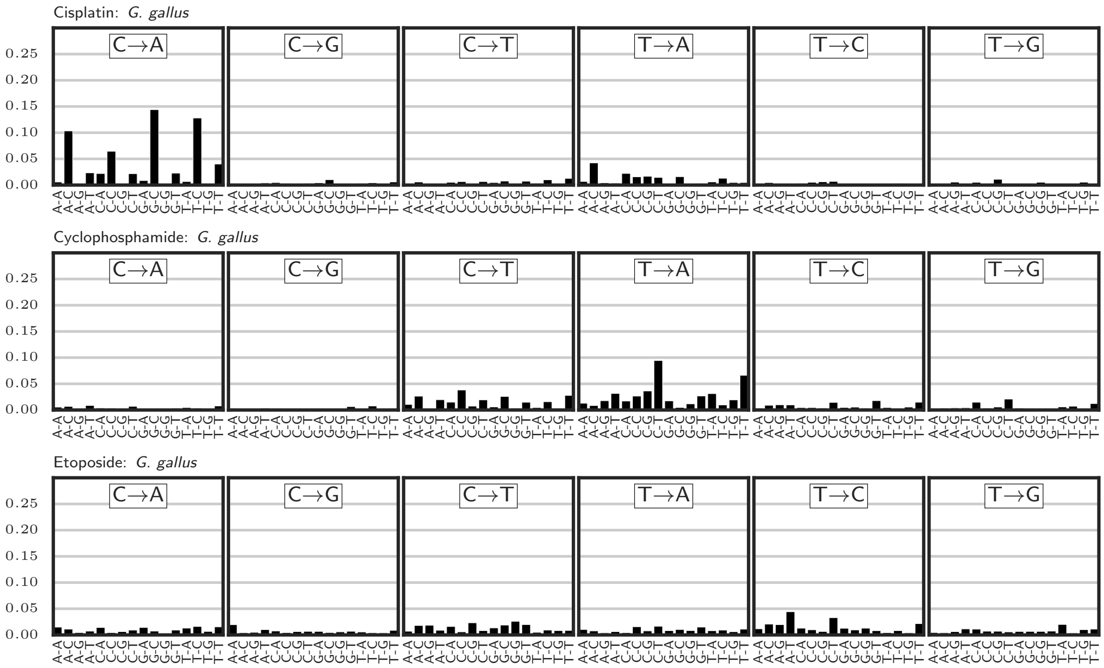

Figure S1: **Mutational signatures extracted from Szikriszt et al.** [1]

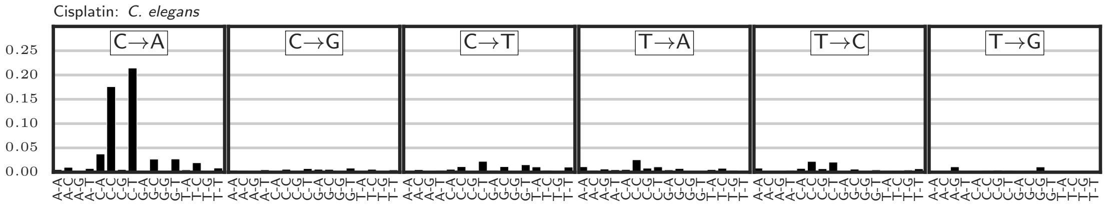

Figure S2: **Mutational signature extracted from Meier et al.** [2]

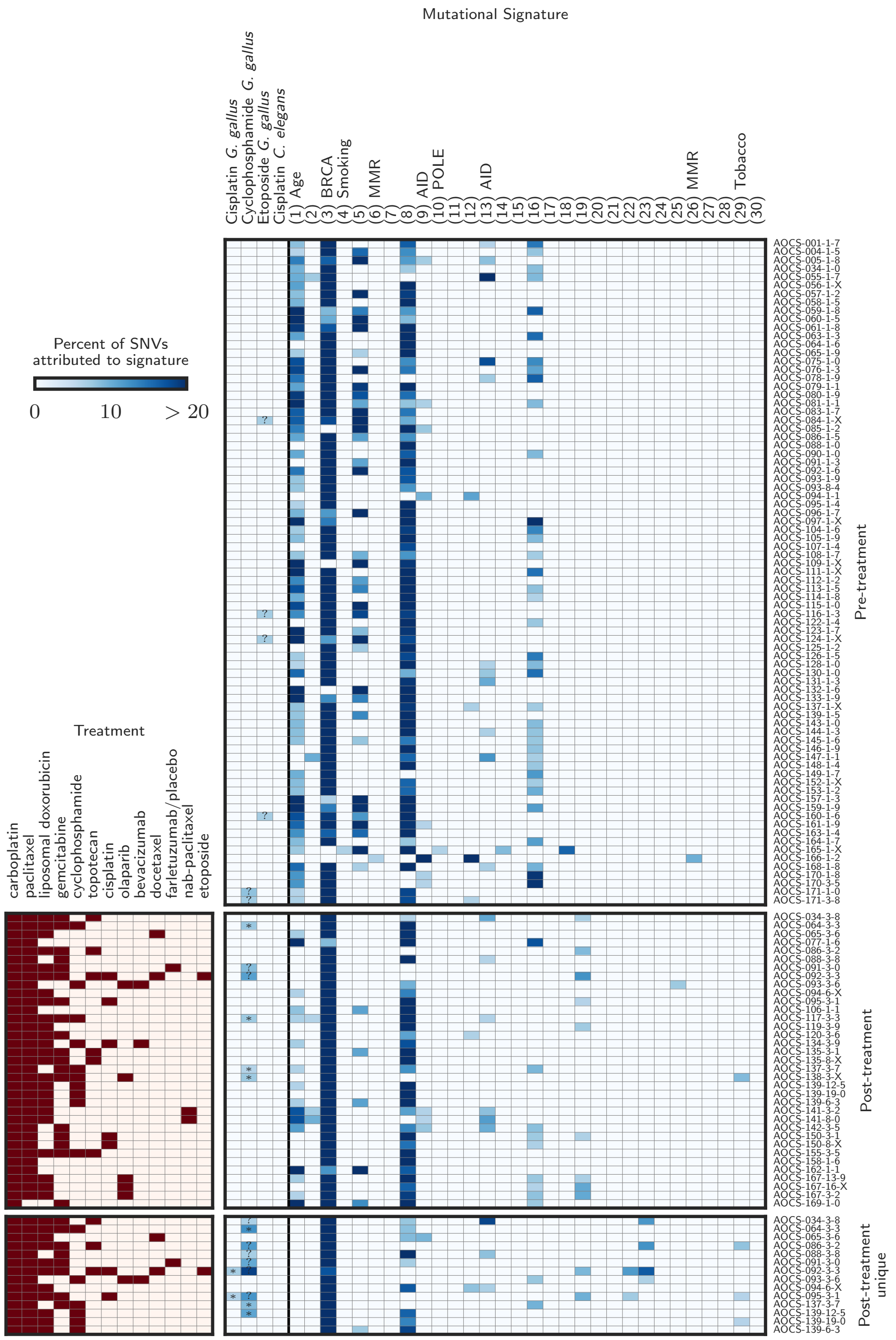

Figure S3: **Detected mutational signatures across all samples.** The symbols are as in main text Figure 1. The top and middle panels show the signature deconvolutions for all pre- and post-treatment samples, respectively. The bottom panel shows deconvolutions for the mutations unique to the paired post-treatment samples, requiring high coverage and no variant reads in the donor-matched pre-treatment sample.

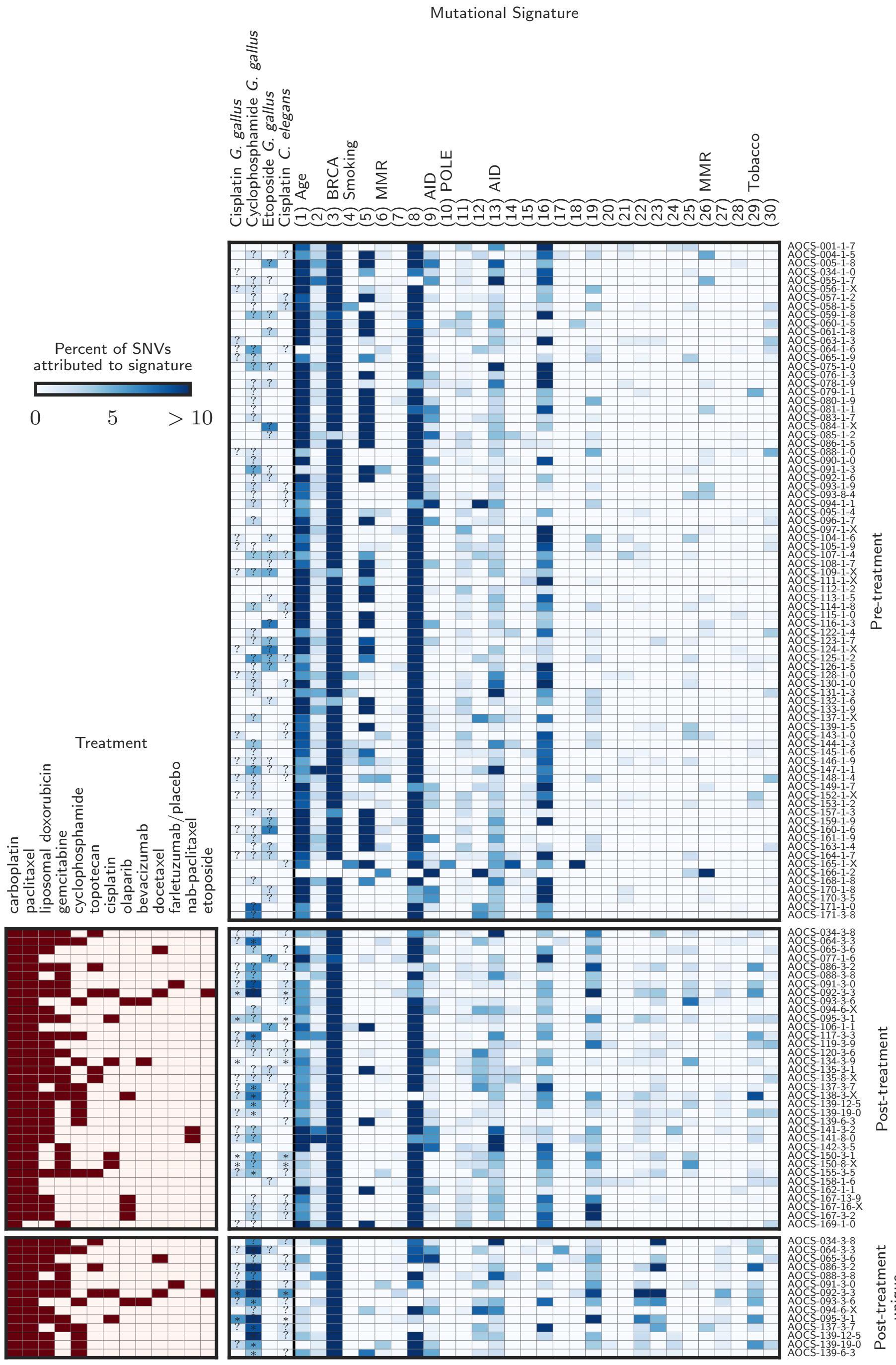

Figure S4: **Mutational signature deconvolutions without any threshold of detection.** Signatures accounting for less than the 6% recommended detection threshold are included.

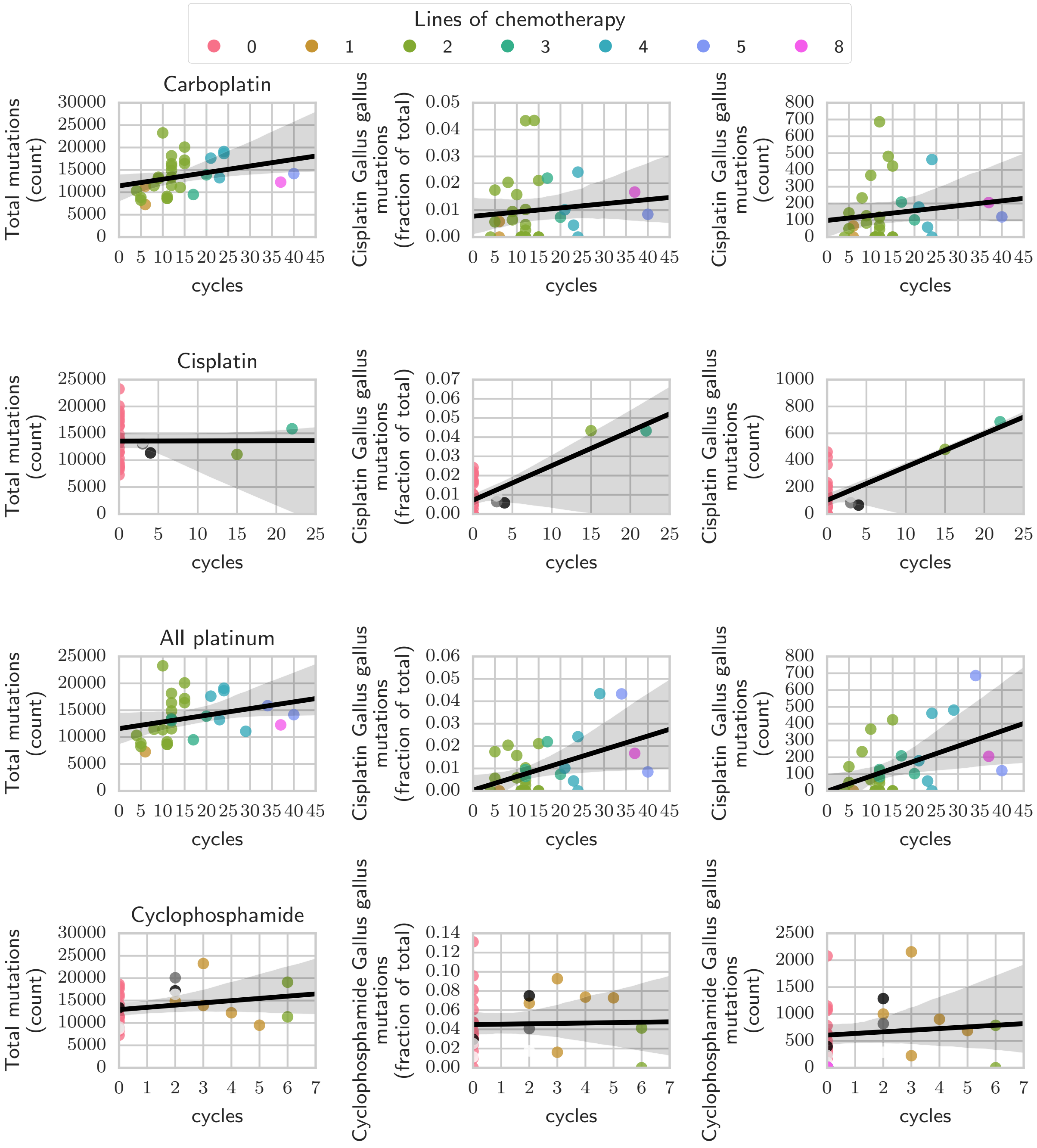

Figure S5: **Association of chemotherapy cycles on total genome-wide mutation burden (left) and mutations attributed to the *G. gallus* cisplatin and cyclophosphamide signatures as a fraction of total (middle) and as a count (right).** The total mutation burden includes both SNVs and indels. Cycles indicated are of the labelled chemotherapy. Colors indicate the number of lines of chemotherapy.

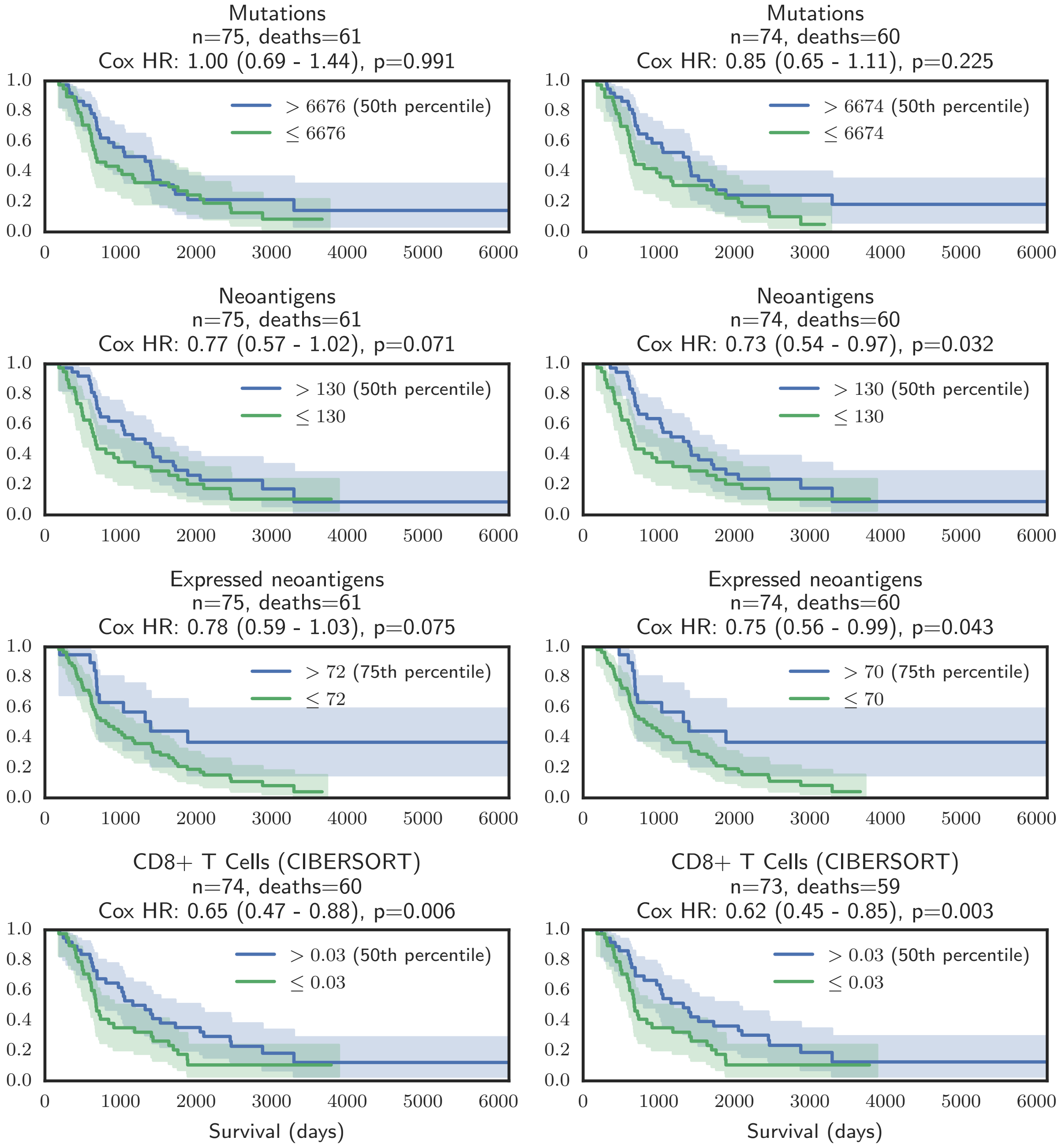

Figure S6: **Kaplan-Meier curves for patients split by the number of mutations, neoantigens, expressed neoantigens, or estimate of CD8+ T cell infiltrate.** Only primary/untreated solid-tissue samples are considered. The survival curves split the samples into high and low groups using a percentile threshold, but the annotated Cox hazard ratio (HR) and p-value correspond to a regression model that treats the value of interest as a continuous covariate. The left plots include all primary/untreated samples; the right plots exclude outlier sample AOCS-166-1-2. The CD8+ T cell analyses exclude sample AOCS-056-1-X, which failed deconvolution.

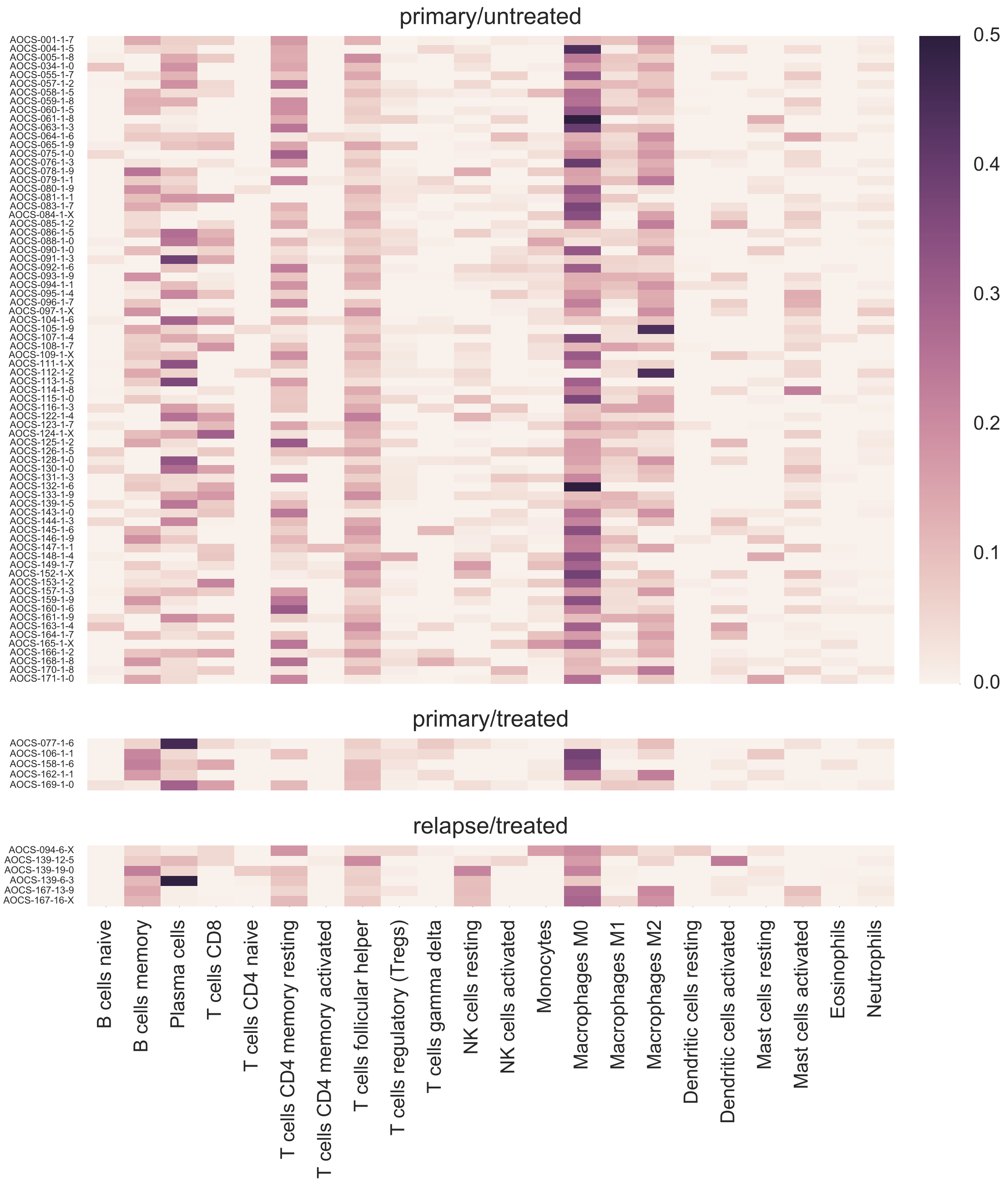

Figure S7: **RNA-seq based immune deconvolution (CIBERSORT) of solid-tissue samples.**

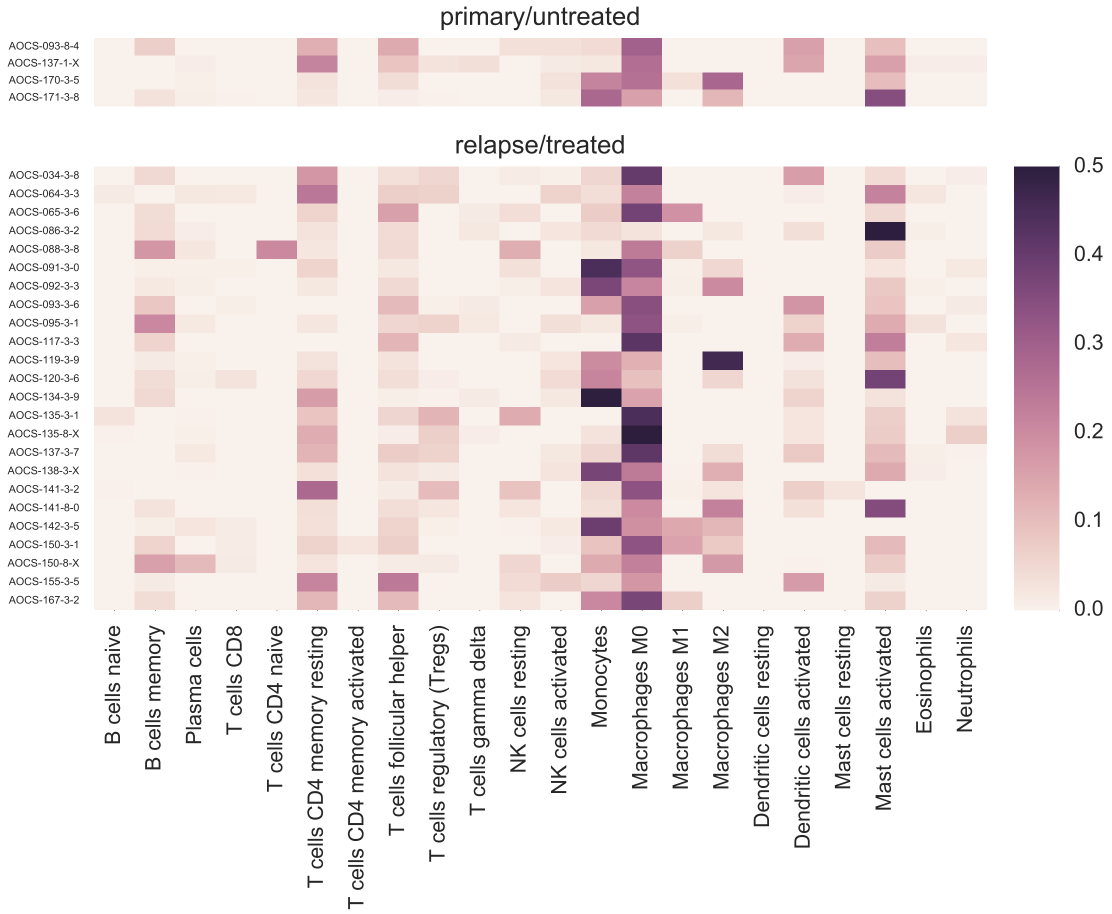

Figure S8: **RNA-seq based immune deconvolution (CIBERSORT) of ascites samples.**

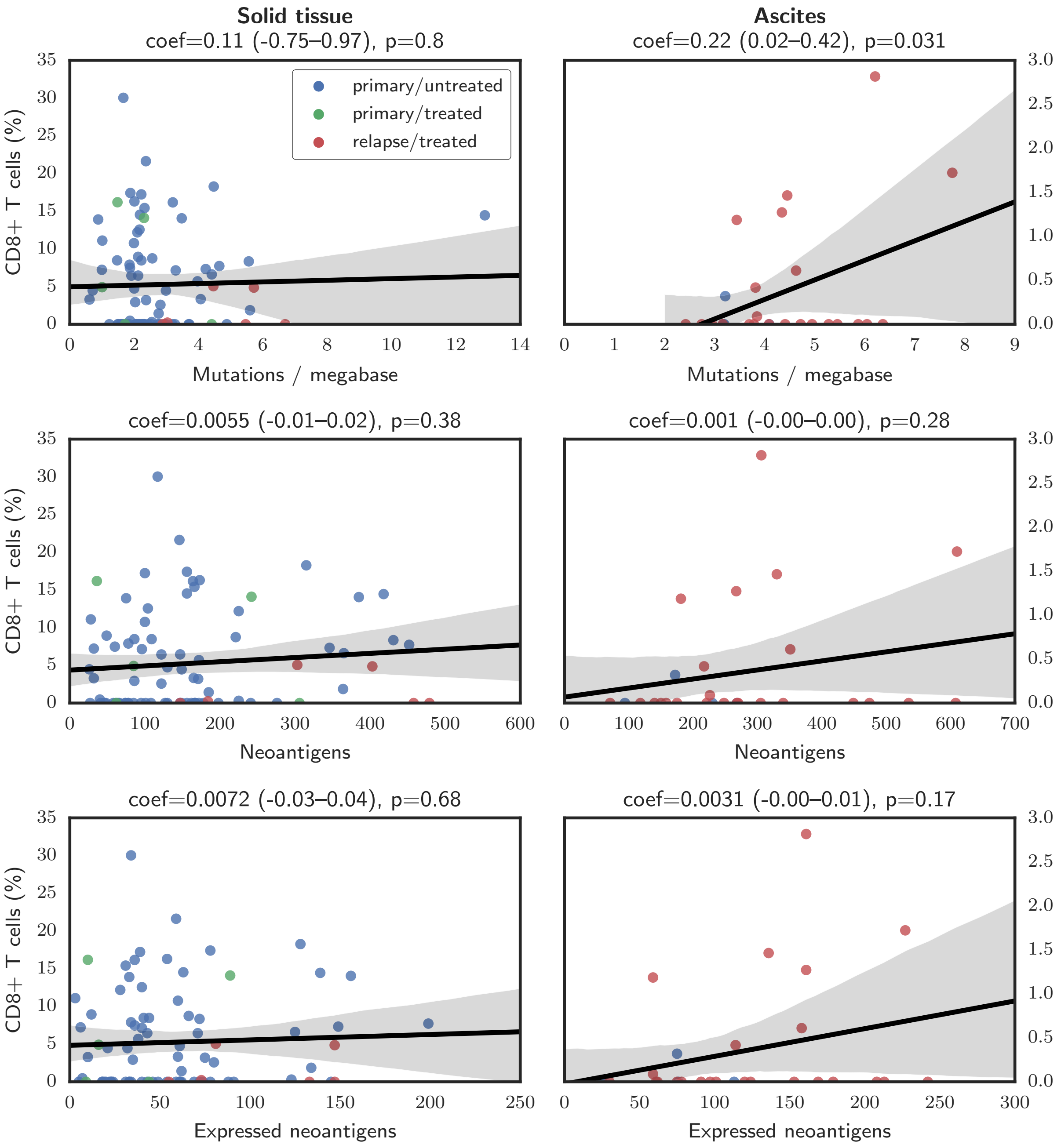

Figure S9: **Relationship between CD8+ T cell infiltrate estimated by CIBERSORT and mutations, neoantigens, or expressed neoantigens for solid tissue (left) and ascites (right) samples.** Colors indicate sample time point.

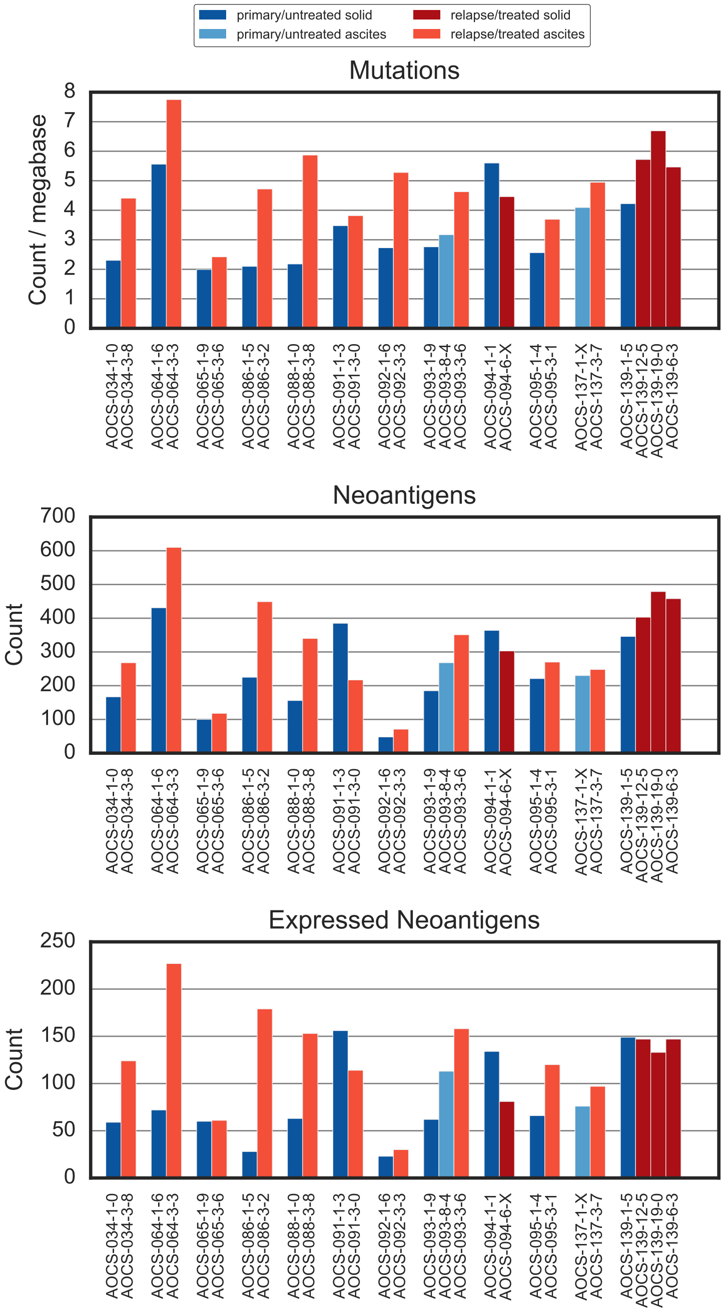

Figure S10: **Mutations, neoantigens, and expressed neoantigens for donor-matched primary/untreated and relapse/treated samples.**

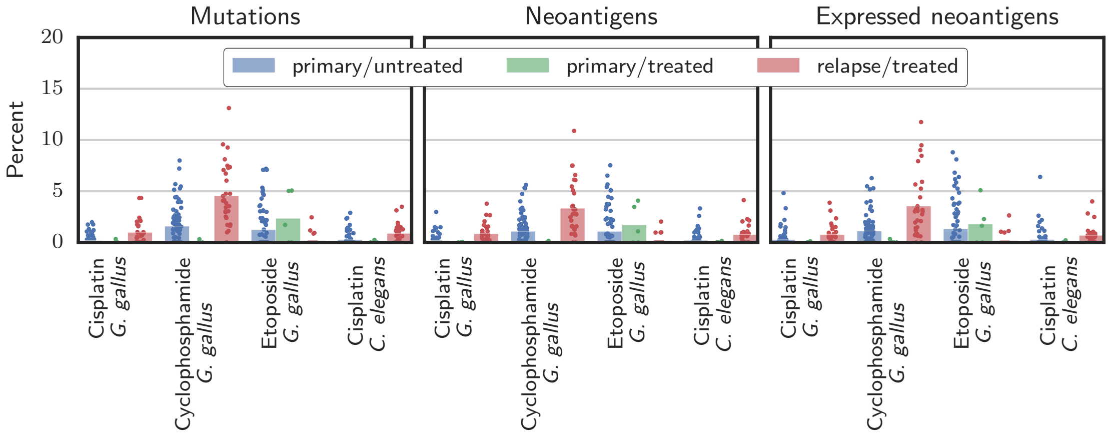

Figure S11: **Contribution of chemotherapy SNV signatures.** The fraction of each sample's mutations, neoantigens, and expressed neoantigens attributed to putative chemotherapy signatures is shown. Bars give the mean, and points indicate individual samples.

References

[1] B. Szikriszt, Á. Póti, O. Pipek, M. Krzystanek, N. Kanu, J. Molnár, D. Ribli, Z. Szeltner, G. E. Tusnády, I. Csabai, Z. Szallasi, C. Swanton, and D. Szts, "A comprehensive survey of the mutagenic impact of common cancer cytotoxics," *Genome Biol*, vol. 17, may 2016.

[2] B. Meier, S. L. Cooke, J. Weiss, A. P. Bailly, L. B. Alexandrov, J. Marshall, K. Raine, M. Maddison, E. Anderson, M. R. Stratton, A. Gartner, and P. J. Campbell, "C. elegans whole-genome sequencing reveals mutational signatures related to carcinogens and DNA repair deficiency," *Genome Research*, vol. 24, pp. 1624–1636, jul 2014.
