# Supplementary material for: Chemotherapy weakly contributes to predicted neoantigen expression in ovarian cancer
Source: BMC Cancer. 2018 Jan 22;18:87. doi: 10.1186/s12885-017-3825-0 (PMC5778667; doi:10.1186/s12885-017-3825-0)
Supplement: Supplementary file 8 — Supplemental statistical notebook. Bayesian models to compare mutation, neoantigen, and expressed neoantigen burdens in treated and untreated samples. (PDF 980 kb) [file 12885_2017_3825_MOESM8_ESM.pdf]

Hierarchical Analysis of Mutation Burden

Jacqueline Buros Novik

7/5/2017

Contents

1 Supplemental Notebook 1 **1**

1.1 Summarize data 1

1.2 Restricting to primary, solid samples 3

1.3 All solid tumor samples 13

1.4 Including ascites samples 20

1.5 Analyzing peptides 29

1 Supplemental Notebook 1

This is a re-analysis of mutation count by sample type (primary / relapse), tissue type (solid vs ascites) and treatment exposure (treatment naive vs treated).

This section uses a Bayesian analysis in order to better adjust for the dramatic imbalance in groups.

1.1 Summarize data

In this analysis we will be looking at how the number of `mutations` & `peptides` (predicted neoantigens) varies with the sample type (solid / ascites) and timing of acquisition (relapse / primary and treated / untreated).

```
ggplot(md, aes(x = mutations_per_mb, fill = specific_treatment)) +
  facet_wrap(~tissue_type) +
  geom_histogram(position = 'dodge') +
  theme_minimal()
```

```
## `stat_bin()` using `bins = 30`. Pick better value with `binwidth`.
```

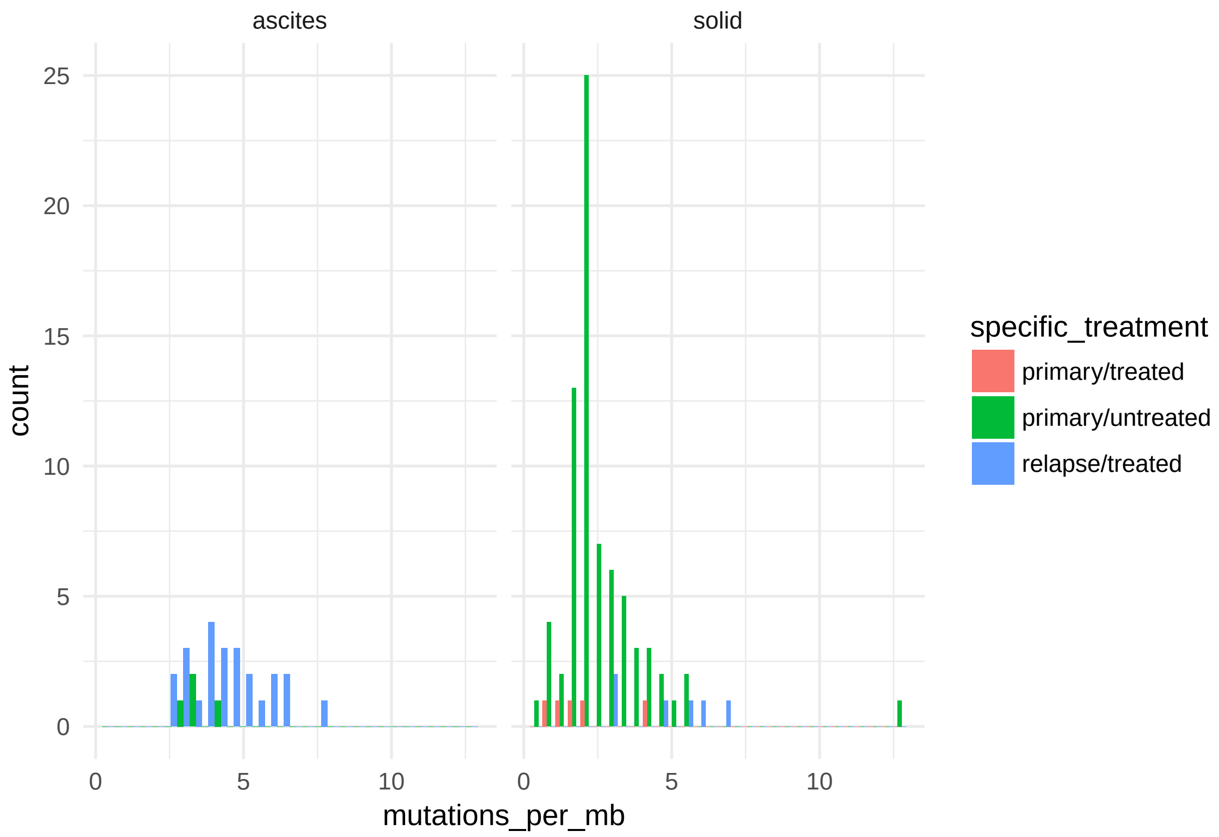

How many observations do we have for each of these categories?

```
md %>%
  group_by(tissue_type, treatment, timepoint) %>%
  tally() %>%
  tidyr::spread(key = tissue_type, value = n, fill = 0)
```

```
## Source: local data frame [3 x 4]
## Groups: treatment [2]
##
##         treatment  timepoint ascites solid
## *           <chr>      <chr>   <dbl> <dbl>
## 1   chemo treated    primary       0     5
## 2   chemo treated recurrence      24     6
## 3 treatment naive    primary       4    75
```

Strikes me that the "recurrent" timepoint is problematic in this analysis, since we don't have any untreated/recurent samples & so cannot separate effect of recurrence from that of treatment.

Instead, we can look at `solid` samples only, among those collected at the primary timepoint comparing treated to untreated samples. My guess is, many of these might be the paired samples.

We can then include the treated / recurrence using only solid samples, although my guess is in this case we will see a higher rate of mutations among recurrence samples than among primary/treated.

Only after these effects are well established should we turn to the ascites samples, to see if the difference between untreated/primary & treated/relapse is consisent with that seen in solid samples.

1.2 Restricting to primary, solid samples

We now have samples, are treatment-naive.

Let's review how these metrics are distributed in this subset of our samples. First we note that the maximum number of samples per donor in this subset of our data is , meaning we have no duplicate samples.

Here, looking at metrics among all primary, solid samples irrespective of treatment:

```
ggplot(md_primary_solid %>%
         tidyr::gather(value = 'value', key = 'variable', mutations, mutations_per_mb, peptides), aes(x
  geom_density() +
  theme_minimal() +
  facet_wrap(~variable, scale = 'free')
```

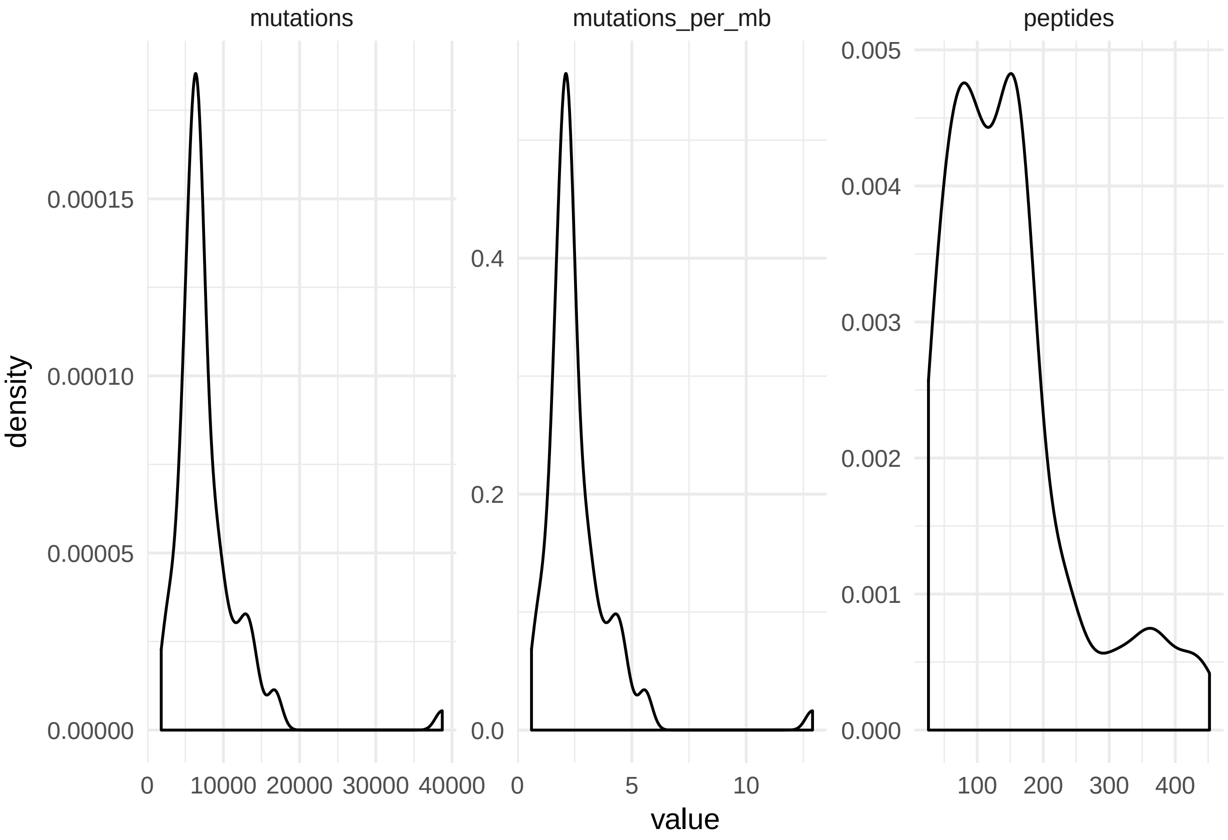

These numbers are not exactly normally-distributed.

Perhaps using a log-transformed value?

```
ggplot(md_primary_solid %>%
         tidyr::gather(value = 'value', key = 'variable', mutations, mutations_per_mb, peptides),
       aes(x = log1p(value))) +
  geom_density() +
  theme_minimal() +
  facet_wrap(~variable, scale = 'free')
```

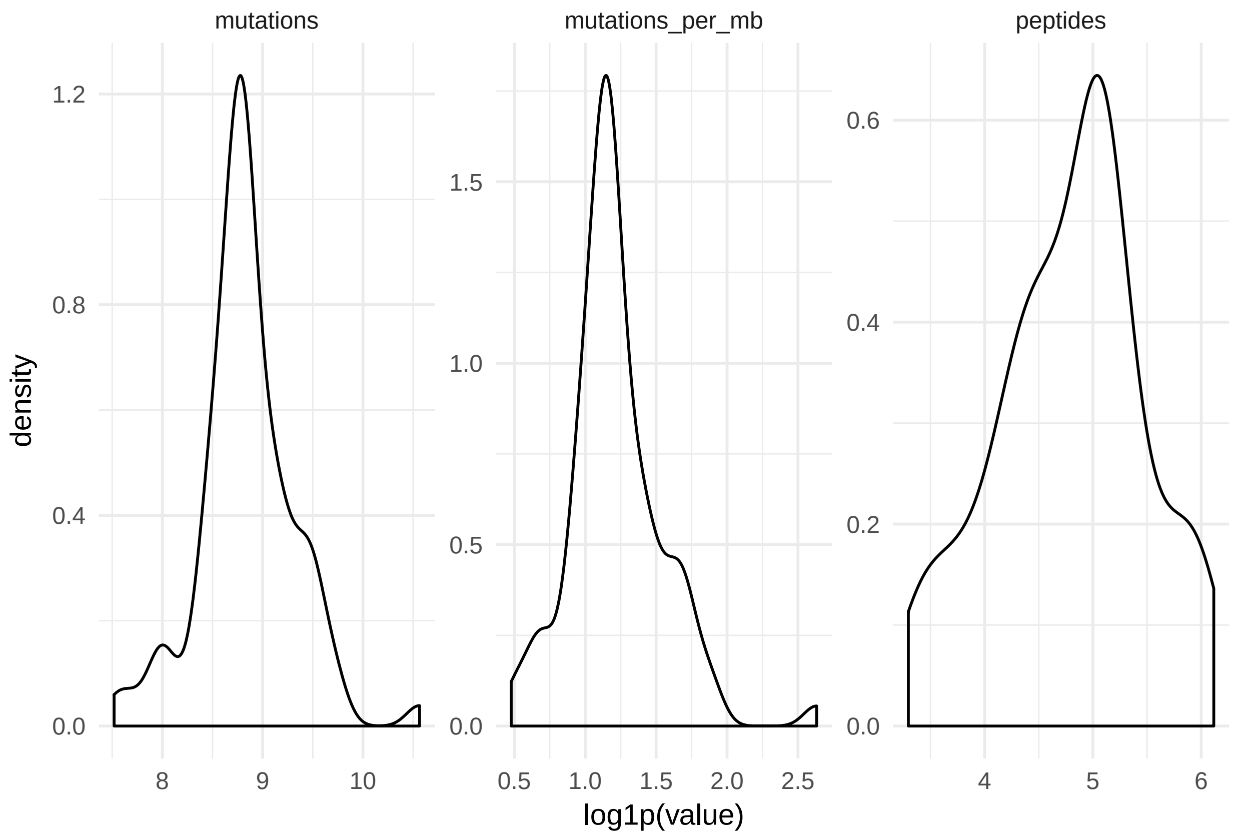


```r
ggplot(md_primary_solid %>%
         tidyr::gather(value = 'value', key = 'variable', mutations, mutations_per_mb, peptides),
       aes(x = log1p(value), fill = treatment)) +
  geom_density(alpha = 0.4) +
  theme_minimal() +
  facet_wrap(~variable, scale = 'free')
```

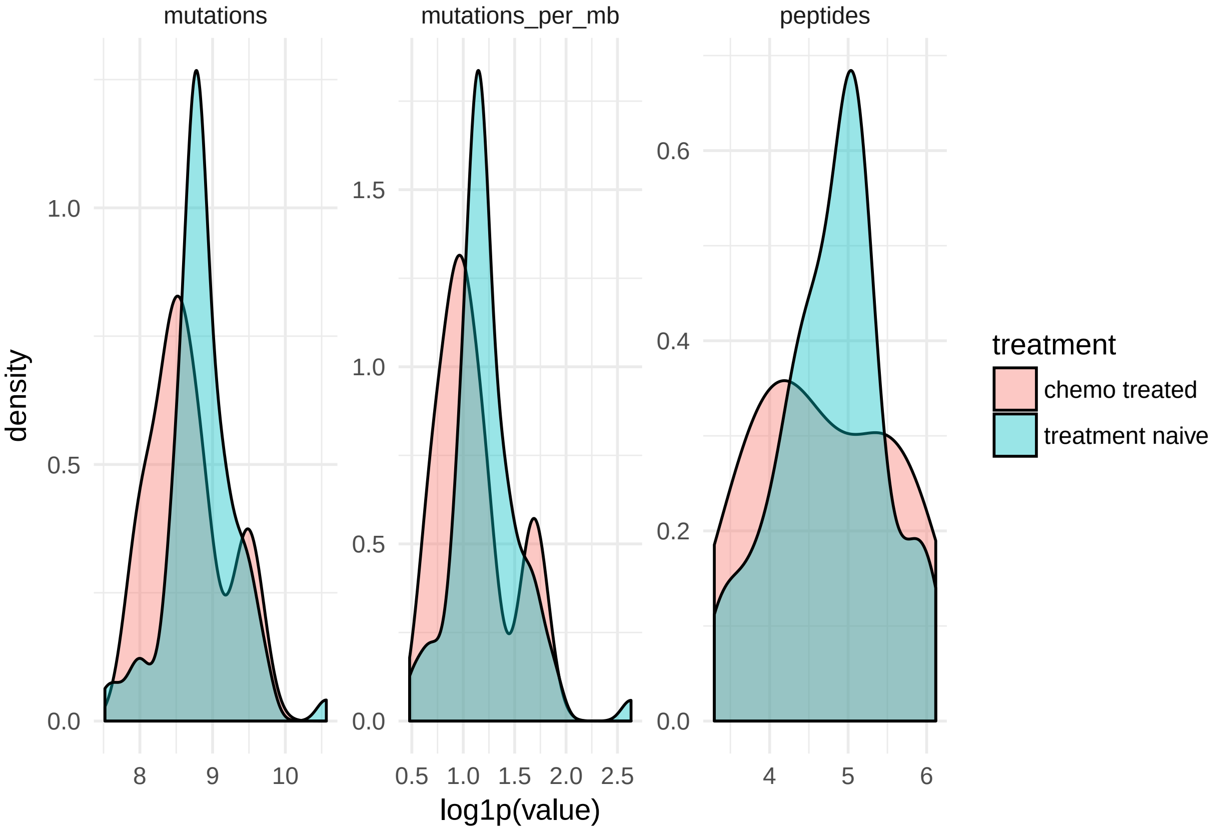

What is noticeable here is that, given the small number of treated samples, it is very hard to tell graphically whether there is any difference in the two distributions.

Let's try fitting a model to these data.

```
trt1 <- rstanarm::stan_glm(log1p(mutations) ~ treatment,
                           data = md_primary_solid,
                           seed = stan_seed
                           )
trt1
```

```
## stan_glm
##  family:  gaussian [identity]
##  formula: log1p(mutations) ~ treatment
## ------
##
## Estimates:
##                            Median MAD_SD
## (Intercept)                8.7    0.2
## treatmenttreatment naive 0.2    0.2
## sigma                      0.5    0.0
##
## Sample avg. posterior predictive
## distribution of y (X = xbar):
##          Median MAD_SD
## mean_PPD 8.8    0.1
##
## ------
```

```
## For info on the priors used see help('prior_summary.stanreg').
```

This suggests the treatment effect on number of mutations may be relatively modest, with a median effect indicating that the average mutation count among treatment naive samples would be 20% higher than that among chemo-treated samples (with a relatively wide posterior interval).

```
bayesplot::mcmc_areas(as.array(trt1), pars = 'treatmenttreatment naive')
```

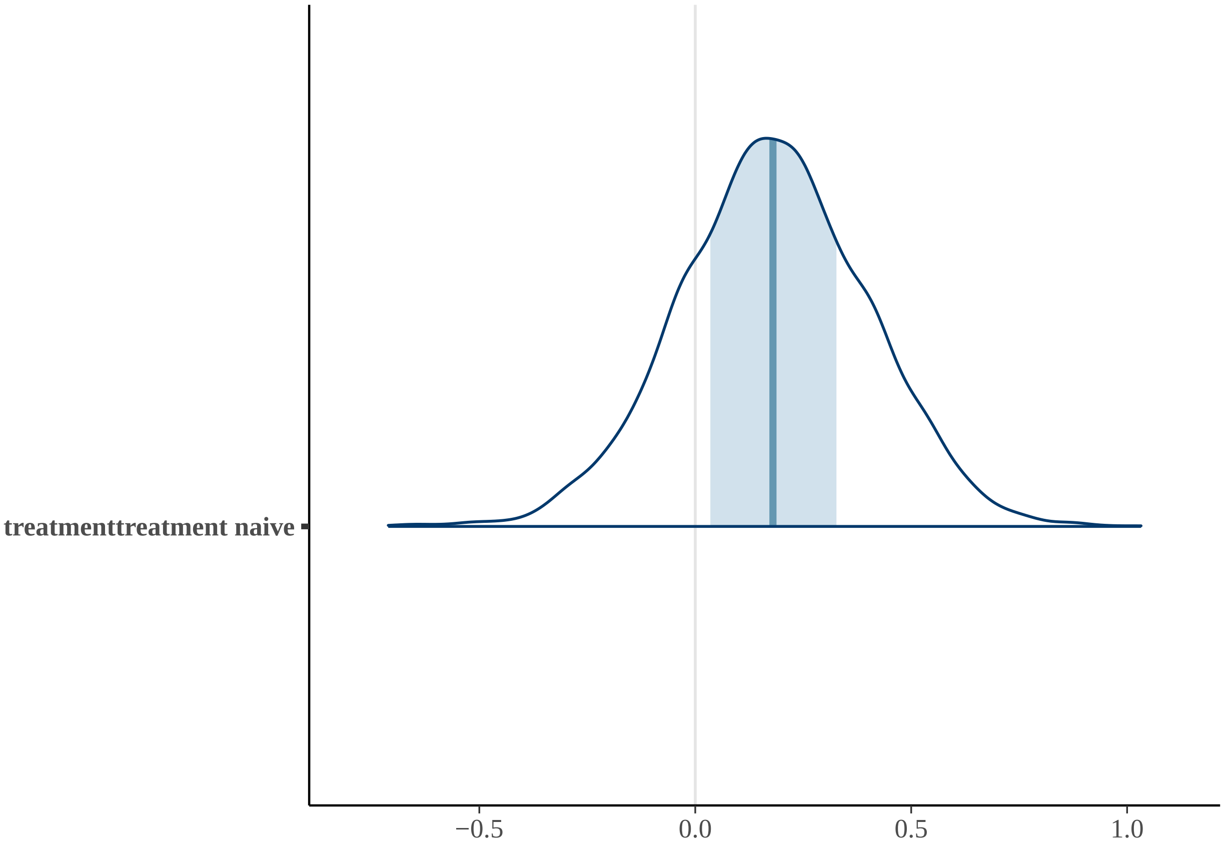

How well do this model's predictions match our data?

```
trt1.ppred <- rstanarm::predictive_interval(trt1) %>%
  tbl_df(.)
trt1.median <- rstanarm::predictive_interval(trt1, 0.01) %>%
  tbl_df(.) %>%
  dplyr::mutate(median = (`49.5%` + `50.5%`)/2) %>%
  dplyr::select(median)

md_primary_solid2 <-
  md_primary_solid %>%
  dplyr::bind_cols(trt1.ppred) %>%
  dplyr::bind_cols(trt1.median)
```

```
ggplot(md_primary_solid2, aes(x = treatment, y = log1p(mutations))) +
  geom_jitter() +
  geom_errorbar(aes(x = treatment, ymin = `5%`, ymax = `95%`),
                data = md_primary_solid2 %>% dplyr::distinct(treatment, .keep_all=T),
                colour = 'red', alpha = 0.5)
```

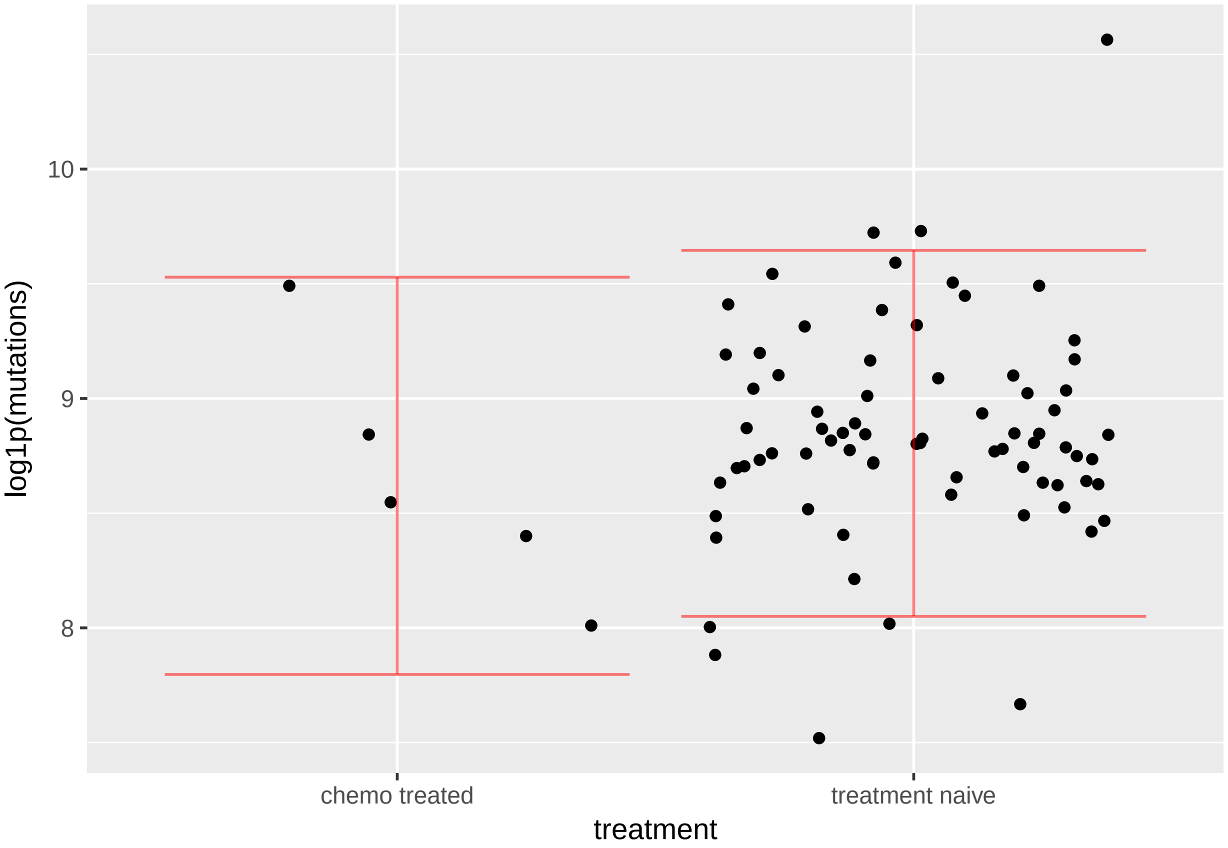

How well does our model recover the observed distributions of variables?

```
bayesplot::pp_check(trt1)
```

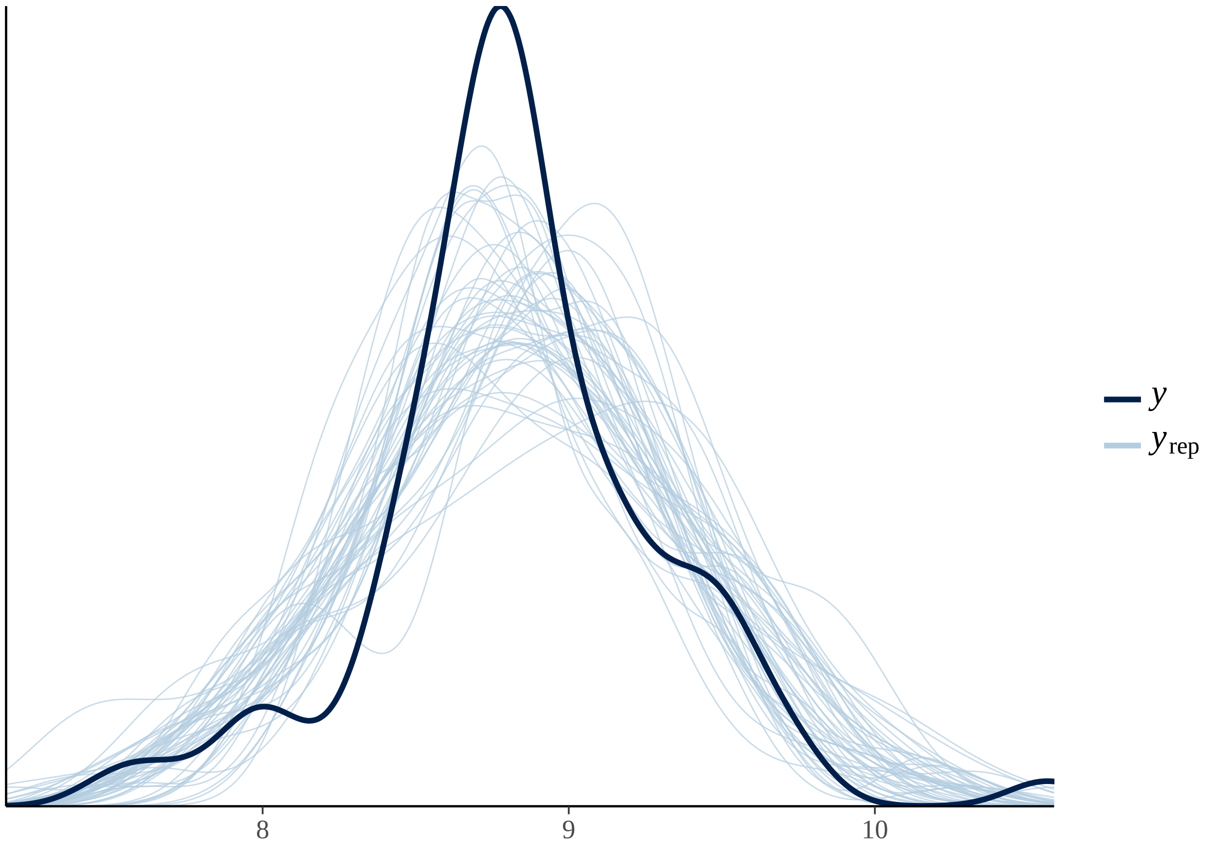

Not bad ..

1.2.1 Try a negative-binomial model?

What if we tried a negative-binomial model instead?

```
trt1nb <- rstanarm::stan_glm(mutations ~ treatment,
                             data = md_primary_solid,
                             family = neg_binomial_2(),
                             seed = stan_seed
)
trt1nb
```

```
## stan_glm
##  family:  neg_binomial_2 [log]
##  formula: mutations ~ treatment
## ------
##
## Estimates:
##                            Median MAD_SD
## (Intercept)                8.8    0.2
## treatmenttreatment naive 0.2    0.2
## reciprocal_dispersion    4.3    0.7
##
## Sample avg. posterior predictive
## distribution of y (X = xbar):
```

```
##          Median MAD_SD
## mean_PPD 7777.7  612.2
##
## ------
## For info on the priors used see help('prior_summary.stanreg').
```

(notice that here we have almost identical parameter estimates)

```
bayesplot::pp_check(trt1nb)
```

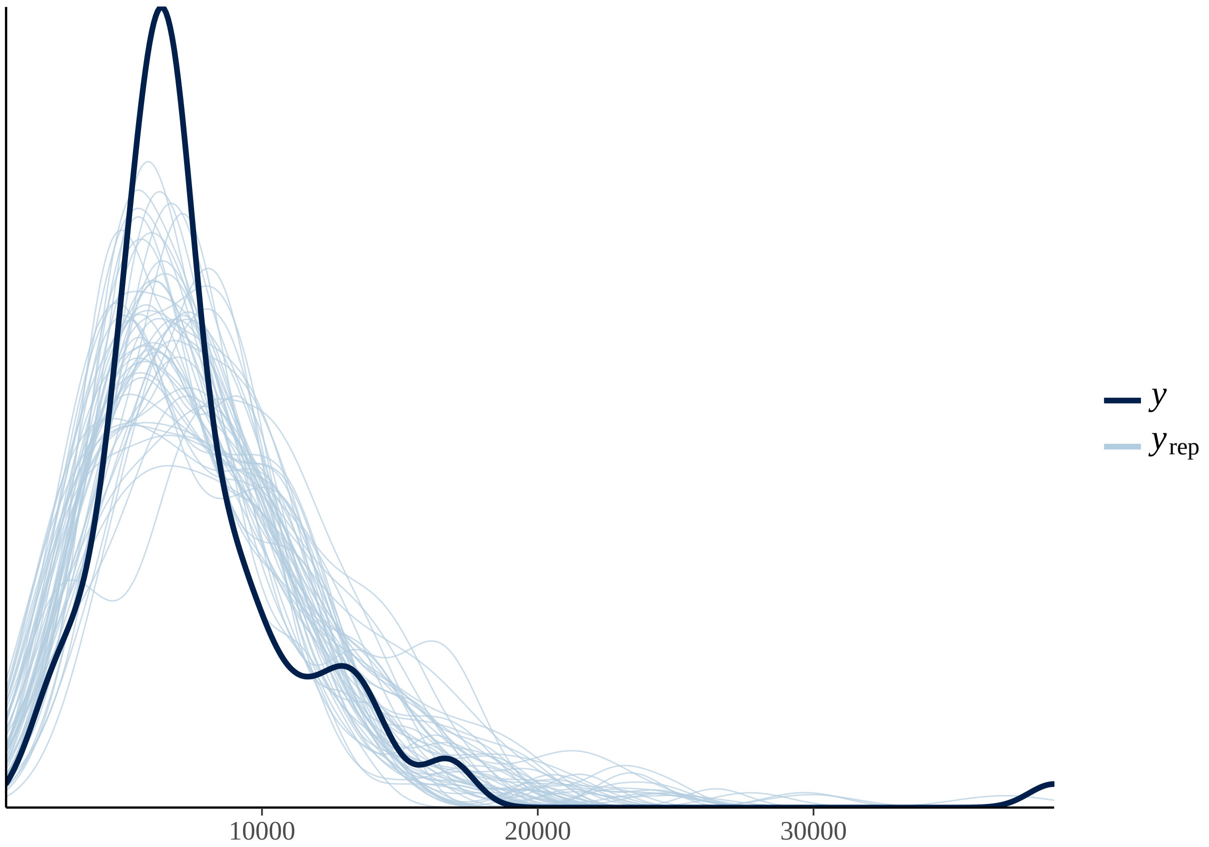

Here we have a slightly better fit, but not by much. Consistent with theory, the log-transform works well as an approximation to the 'counting process' at high levels of the counts.

1.2.2 Adjust for number of cycles?

Next we look at estimating the effects of number of cycles on mutation count. Sometimes adding more information can address noise in the model, and sometimes it just .. adds noise.

```
trt2 <- rstanarm::stan_glm(log1p(mutations) ~ treatment + `total cycles`,
                           data = md_primary_solid %>%
                             dplyr::mutate(no_treatment = ifelse(treatment == 'treatment naive', 1, 0),
                                           treatment = ifelse(treatment != 'treatment naive', 1, 0))
                           )
trt2
```

```
## stan_glm
```

```
##  family:  gaussian [identity]
##  formula: log1p(mutations) ~ treatment + `total cycles`
## ------
##
## Estimates:
##                 Median MAD_SD
## (Intercept)     8.9    0.1
## treatment       1.1    0.7
## `total cycles` -0.2    0.1
## sigma           0.5    0.0
##
## Sample avg. posterior predictive
## distribution of y (X = xbar):
##          Median MAD_SD
## mean_PPD 8.8    0.1
##
## ------
## For info on the priors used see help('prior_summary.stanreg').
```

Let's plot the distributions around these effects

```
bayesplot::mcmc_areas(as.array(trt2), pars = c('treatment', '`total cycles`'))
```

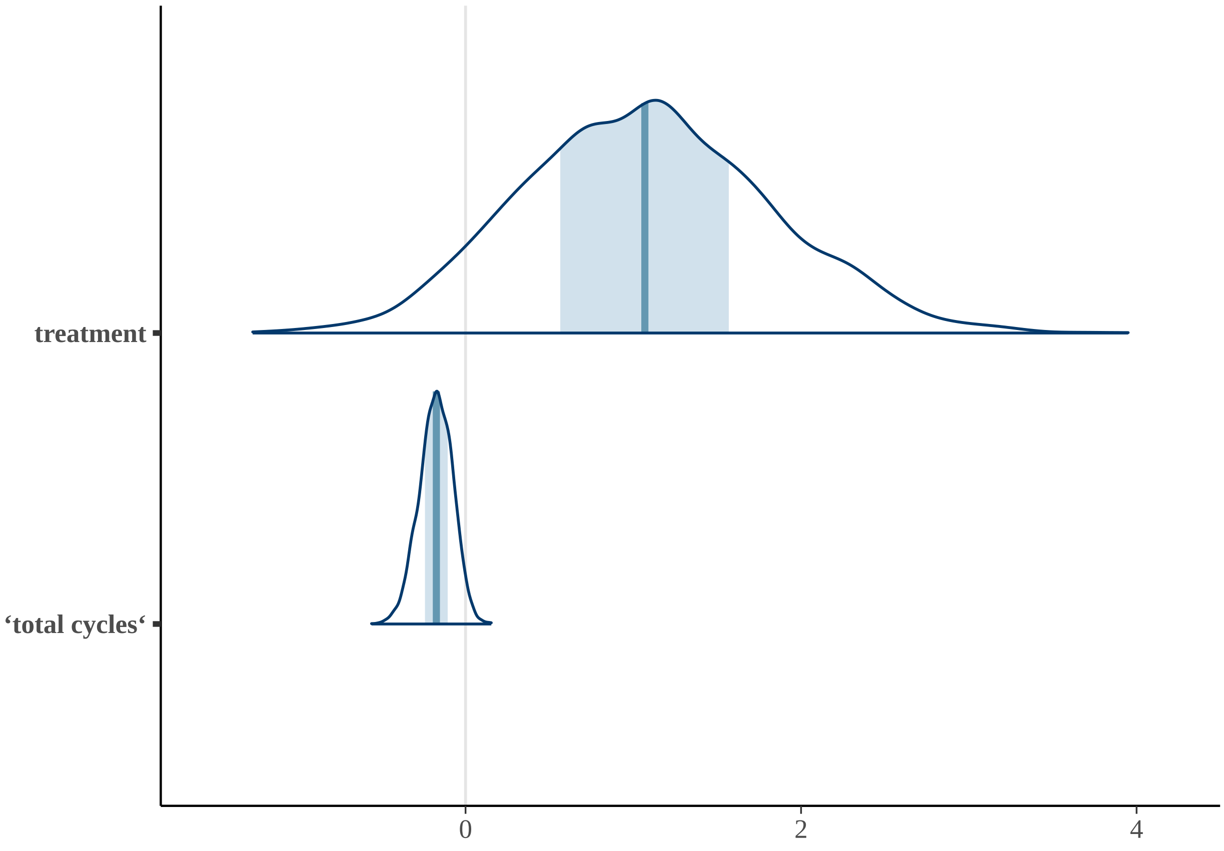

The interpretation of these results would be that :

1. Samples that received treatment have higher average mutation count than samples that are treatment-naive

2. Among those receiving treatment, those with more cycles tended to have lower mutation count

This may or may not make biological sense (to me it feels like a stretch), and the posterior distributions of effects are all pretty broad. So my inclination would be to judge these effects as being "within the noise".

1.2.3 Use a varying-intercept effect?

Before moving on to include solid/relapse/treated & ascites samples, we fit the same model using a varying-coefficient structure. We do this because this is the type of model we will fit in later iterations and so it will be helpful to have a baseline here to compare against.

This model is structurally very similar to fitting a no-intercept model, with formula `mutations ~ 0 + treatment`, which would estimate separate mean numbers of mutations among treated & naive solid primary samples. The difference is that, in this formulation, the treatment-specific means are drawn from a higher-level distribution of means which acts like a prior for the group-specific means, and which in effect regularizes the treatment & non-treatment means – the group-specific means end up shrinking towards an overall inter-group mean.

```
trt3 <- rstanarm::stan_glmer(log1p(mutations) ~ (1 | treatment),
                           data = md_primary_solid,
                           adapt_delta = 0.999,
                           seed = stan_seed
                           )
trt3
```

```
## stan_glmer
##  family:  gaussian [identity]
##  formula: log1p(mutations) ~ (1 | treatment)
## ------
##
## Estimates:
##             Median MAD_SD
## (Intercept) 8.8    0.2
## sigma       0.5    0.0
##
## Error terms:
##  Groups    Name        Std.Dev.
##  treatment (Intercept) 0.42
##  Residual              0.48
## Num. levels: treatment 2
##
## Sample avg. posterior predictive
## distribution of y (X = xbar):
##          Median MAD_SD
## mean_PPD 8.8    0.1
##
## ------
## For info on the priors used see help('prior_summary.stanreg').
```

The summary for this model does not include the group-specific means by default. We can, however, recover them quite easily.

```r
bayesplot::mcmc_areas(as.array(trt3), regex_pars = '\\(Intercept\\) treatment\\:')
```

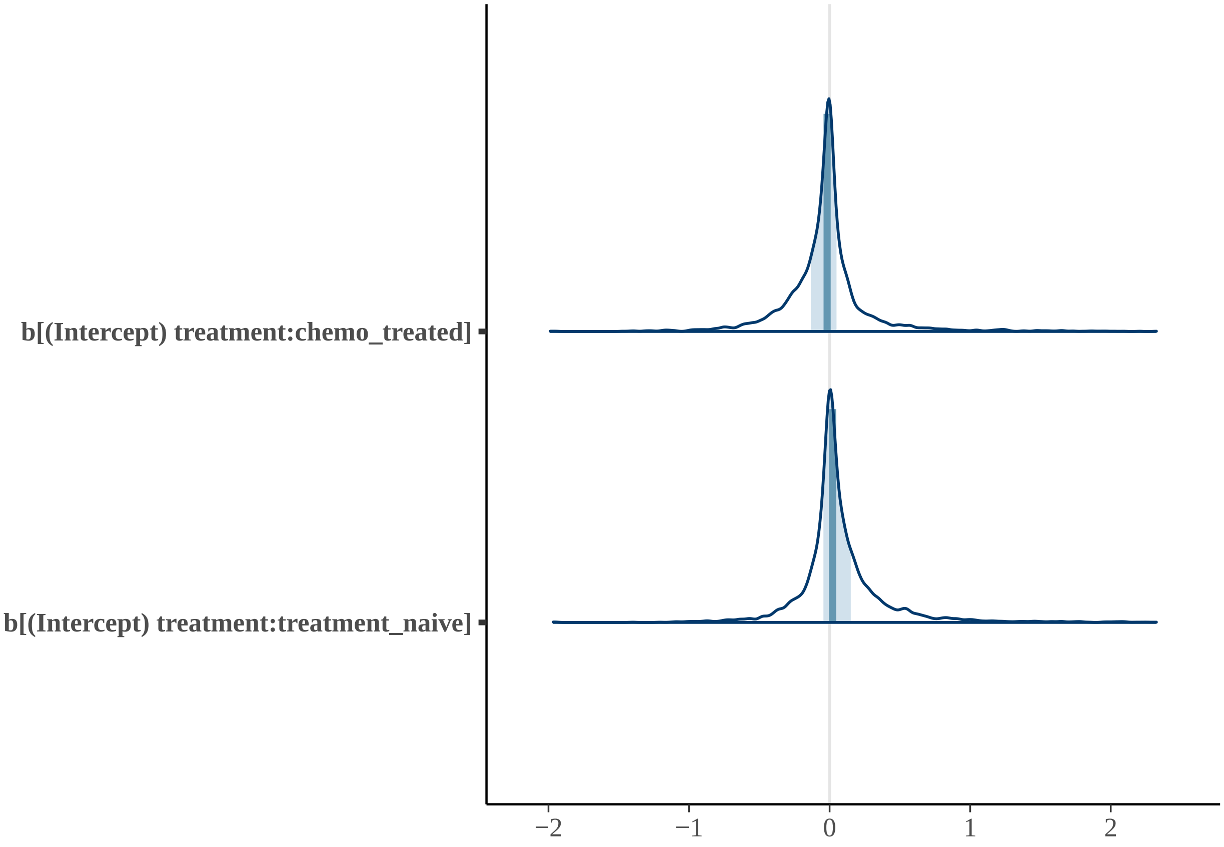

A textual summary can be accessed via `summary`:

```r
summary(trt3, regex_pars = c('^b'))
```

```
##
## Model Info:
##
##  function:  stan_glmer
##  family:    gaussian [identity]
##  formula:   log1p(mutations) ~ (1 | treatment)
##  algorithm: sampling
##  priors:    see help('prior_summary')
##  sample:    4000 (posterior sample size)
##  num obs:   80
##  groups:    treatment (2)
##
## Estimates:
##                                             mean   sd   2.5%   25%   50%
## b[(Intercept) treatment:chemo_treated]    0.0    0.3 -0.6   -0.1   0.0
## b[(Intercept) treatment:treatment_naive]  0.1    0.3 -0.5    0.0   0.0
##                                             75%   97.5%
## b[(Intercept) treatment:chemo_treated]    0.0   0.7
## b[(Intercept) treatment:treatment_naive]  0.2   0.9
##
```

```
## Diagnostics:
##                                          mcse Rhat n_eff
## b[(Intercept) treatment:chemo_treated]   0.0  1.0  730
## b[(Intercept) treatment:treatment_naive] 0.0  1.0  594
##
## For each parameter, mcse is Monte Carlo standard error, n_eff is a crude measure of effective sample
```

However, in order to estimate the average difference between treated and naive samples, it is easiest to use posterior-predicted values.

For this, we construct a hypothetical dataset containing one treated & one treatment-naive sample.

```
newdata <- data.frame(treatment = c('chemo_treated', 'treatment_naive'),
                      timepoint = c('primary', 'primary'))
ppred_newdata <- rstanarm::posterior_predict(trt3, newdata = newdata)
summary(apply(ppred_newdata, 1, diff))
```

```
##     Min.  1st Qu.   Median     Mean  3rd Qu.     Max.
## -2.43100 -0.36080  0.09884  0.10520  0.57850  2.61800
```

This yields the posterior-predicted distribution of the difference (on scale of `log1(mutations)`) between chemo-treated & treatment-naive samples.

It's not surprising, here, that our effect has shrunken – the mean effect of having a treatment_naive sample is 0.12 (or 12% higher mutation count than chemo-treated samples) instead of 0.2. Our confidence intervals around this difference is also much greater than in the previous model. However the direction of effect is similar. Folks may reasonably disagree about which of these two results is more "correct", but the findings aren't inconsistent.

At this point, we are ready to move on to a variation of this model that includes all solid samples, estimating effects of relapse & treatment separately.

1.3 All solid tumor samples

Now we start to analyze a dataset including primary/untreated, primary/treated & relapse/treated samples.

First we note that the maximum number of samples per donor in this subset of our data is , meaning we have a handful of duplicate samples per donor. We will adjust for this in our analysis.

1.3.1 Using a standard Bayesian glm

First we fit a standard `glm` without any donor-specific adjustments.

```
strt1 <- rstanarm::stan_glm(log1p(mutations) ~ treatment + timepoint,
                            data = md_solid,
                            adapt_delta = 0.999,
                            seed = stan_seed
                            )
strt1
```

```
## stan_glm
##  family:  gaussian [identity]
##  formula: log1p(mutations) ~ treatment + timepoint
```

```
## ------
##
## Estimates:
##                           Median MAD_SD
## (Intercept)               8.7    0.2
## treatmenttreatment naive 0.2    0.2
## timepointrecurrence      0.8    0.3
## sigma                     0.5    0.0
##
## Sample avg. posterior predictive
## distribution of y (X = xbar):
##          Median MAD_SD
## mean_PPD 8.9    0.1
##
## ------
## For info on the priors used see help('prior_summary.stanreg').
```

Here, we see a similar treatment effect as in our earlier analysis (which is encouraging), but the estimated "recurrence" effect is somewhat higher than the treatment effect.

How well are we recovering the distribution of our `log1p(mutation)` count in this sample?

```
bayesplot::pp_check(strt1)
```

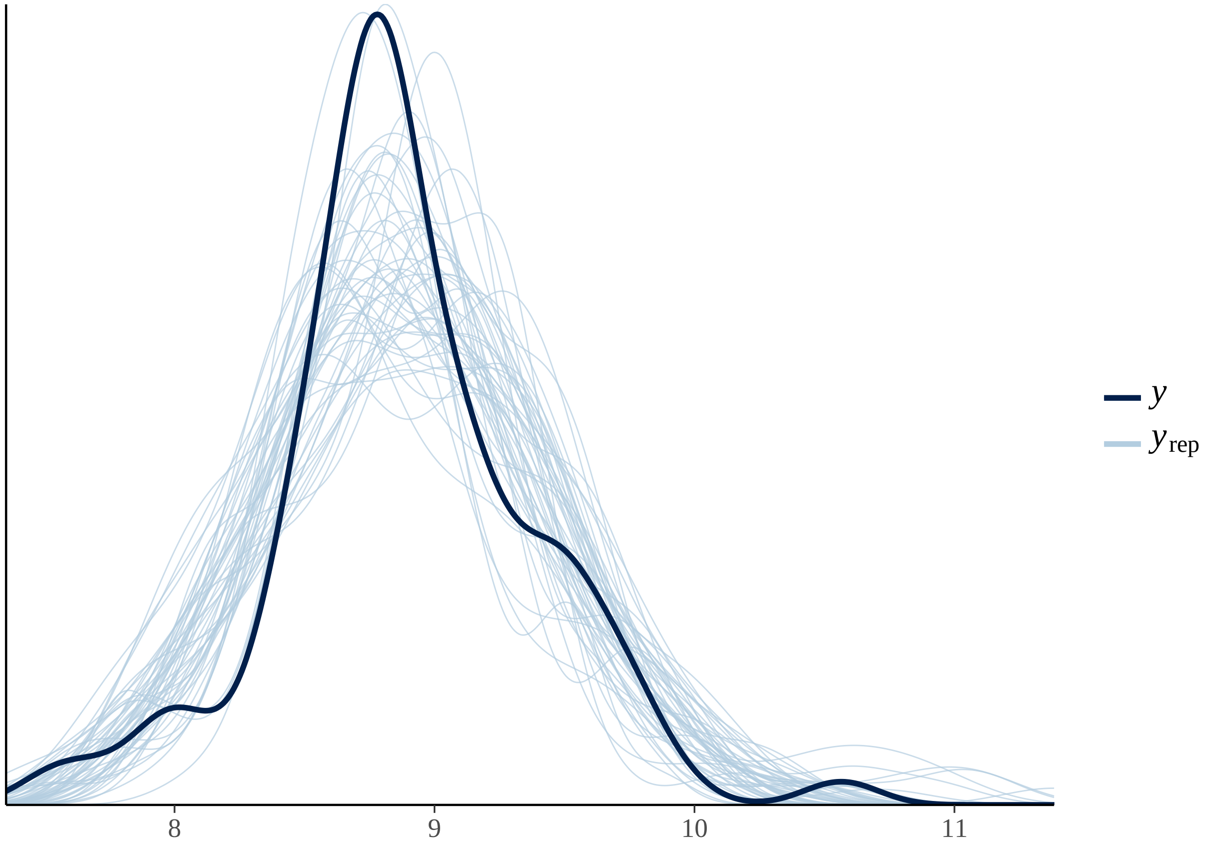

Pretty well.

1.3.2 Using a varying-intercept model

Let's fit this model with an adjustment for within-id similarity.

```
strt2 <- rstanarm::stan_glmer(log1p(mutations) ~ treatment + timepoint + (1 | donor),
                              data = md_solid,
                              adapt_delta = 0.999,
                              iter = 5000,
                              seed = stan_seed
                              )
```

```
## Warning: There were 4 chains where the estimated Bayesian Fraction of Missing Information was low. Se
## http://mc-stan.org/misc/warnings.html#bfmi-low
```

```
## Warning: Examine the pairs() plot to diagnose sampling problems
```

```
strt2
```

```
## stan_glmer
##  family:  gaussian [identity]
##  formula: log1p(mutations) ~ treatment + timepoint + (1 | donor)
## ------
##
## Estimates:
##                          Median MAD_SD
## (Intercept)              8.7    0.2
## treatmenttreatment naive 0.2    0.2
## timepointrecurrence      0.5    0.3
## sigma                    0.3    0.1
##
## Error terms:
##  Groups   Name        Std.Dev.
##  donor    (Intercept) 0.36
##  Residual             0.29
## Num. levels: donor 81
##
## Sample avg. posterior predictive
## distribution of y (X = xbar):
##          Median MAD_SD
## mean_PPD 8.9    0.0
##
## ------
## For info on the priors used see help('prior_summary.stanreg').
```

These findings are similar to those from the earlier model which did not adjust for duplicate samples per donor, although the effect is somewhat attenuated.

```
bayesplot::mcmc_areas(as.array(strt2), regex_pars = c('^treatment', '^timepoint'))
```

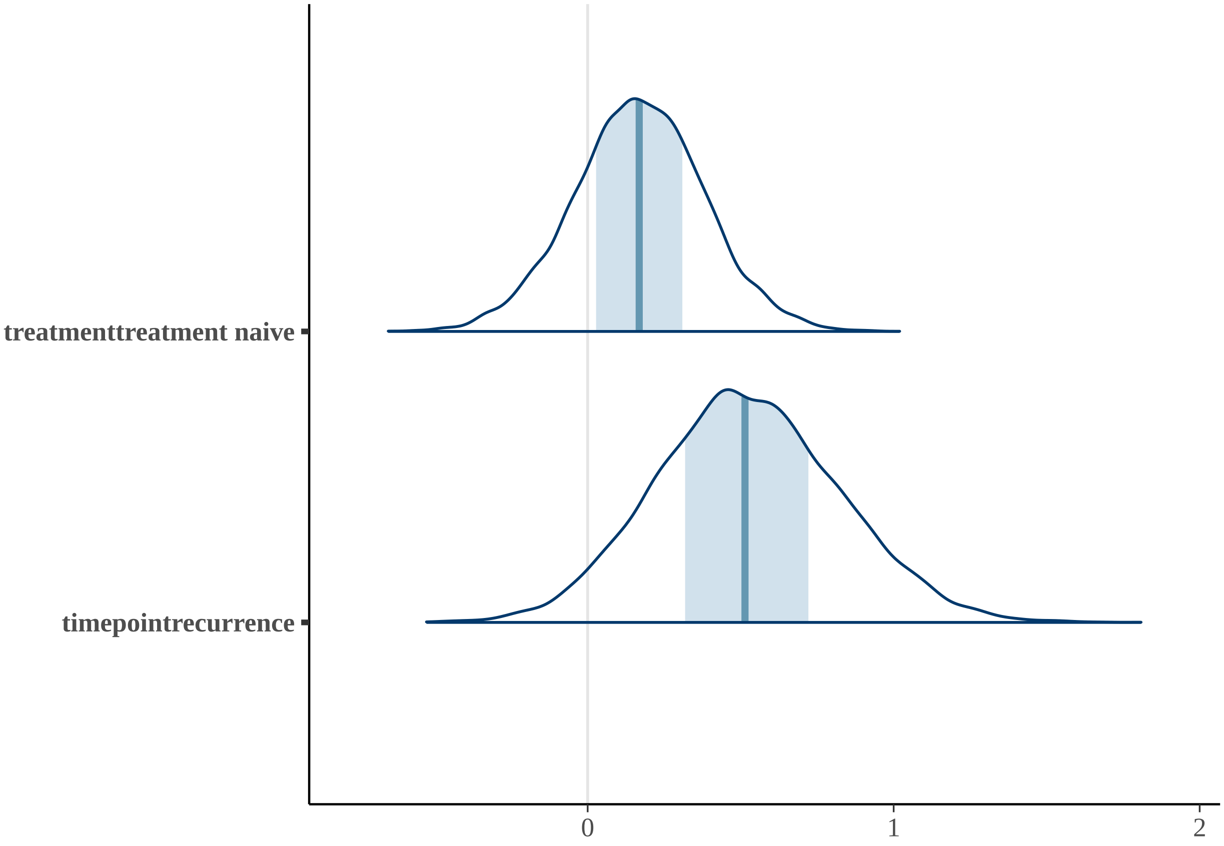

According to this model, we have an estimated average 50% increase in mutations for relapse samples, vs primary.

The 95% credible intervals for this increase are:

```
rstanarm::posterior_interval(strt2, prob = 0.95, pars = c('timepointrecurrence'))
```

```
##                            2.5%    97.5%
## timepointrecurrence -0.05066051 1.125876
```

How much of this probability mass is < 0?

```
mean(sapply(as.array(strt2)[,,'timepointrecurrence'], FUN = function(x) x <= 0))
```

```
## [1] 0.0383
```

This number reflects the so-called 'bayesian p-value', IE the posterior probability of a relapse effect being <= 0. Note that this would correspond to a one-sided p-value in traditional frequentist NHT framework.

Side note: this adjustment for `donor` only accounts for the fact that we'd expect two samples from the same donor to be more similar from one another than two samples from different donors. It does not estimate "varying-coefficients", ie relapse or treatment effects cannot vary by donor. All effects are estimated as population averages.

1.3.3 Using a varying-coefficient model

Next we fit a varying-coefficient model, which allows the relapse effect to vary by donor, but models those donor-specific variances as deviations from an overall relapse effect on mutation count.

```
strt3 <- rstanarm::stan_glmer(log1p(mutations) ~ treatment + timepoint + (1 + timepoint | donor),
                              data = md_solid,
                              adapt_delta = 0.999,
                              seed = stan_seed
                              )
```

```
## Warning: There were 4 chains where the estimated Bayesian Fraction of Missing Information was low. Se
## http://mc-stan.org/misc/warnings.html#bfmi-low
```

```
## Warning: Examine the pairs() plot to diagnose sampling problems
```

```
strt3
```

```
## stan_glmer
##  family:  gaussian [identity]
##  formula: log1p(mutations) ~ treatment + timepoint + (1 + timepoint | donor)
## ------
##
## Estimates:
##                          Median MAD_SD
## (Intercept)              8.7    0.2
## treatmenttreatment naive 0.2    0.2
## timepointrecurrence      0.6    0.4
## sigma                    0.3    0.2
##
## Error terms:
##  Groups   Name                Std.Dev. Corr
##  donor    (Intercept)         0.33
##           timepointrecurrence 0.31     -0.09
##  Residual                     0.32
## Num. levels: donor 81
##
## Sample avg. posterior predictive
## distribution of y (X = xbar):
##          Median MAD_SD
## mean_PPD 8.9    0.0
##
## ------
## For info on the priors used see help('prior_summary.stanreg').
```

As in our previous examples, we can plot the posterior density of the estimated treatment & relapse effects.

```
bayesplot::mcmc_areas(as.array(strt3), regex_pars = c('^treatment', '^timepoint'))
```

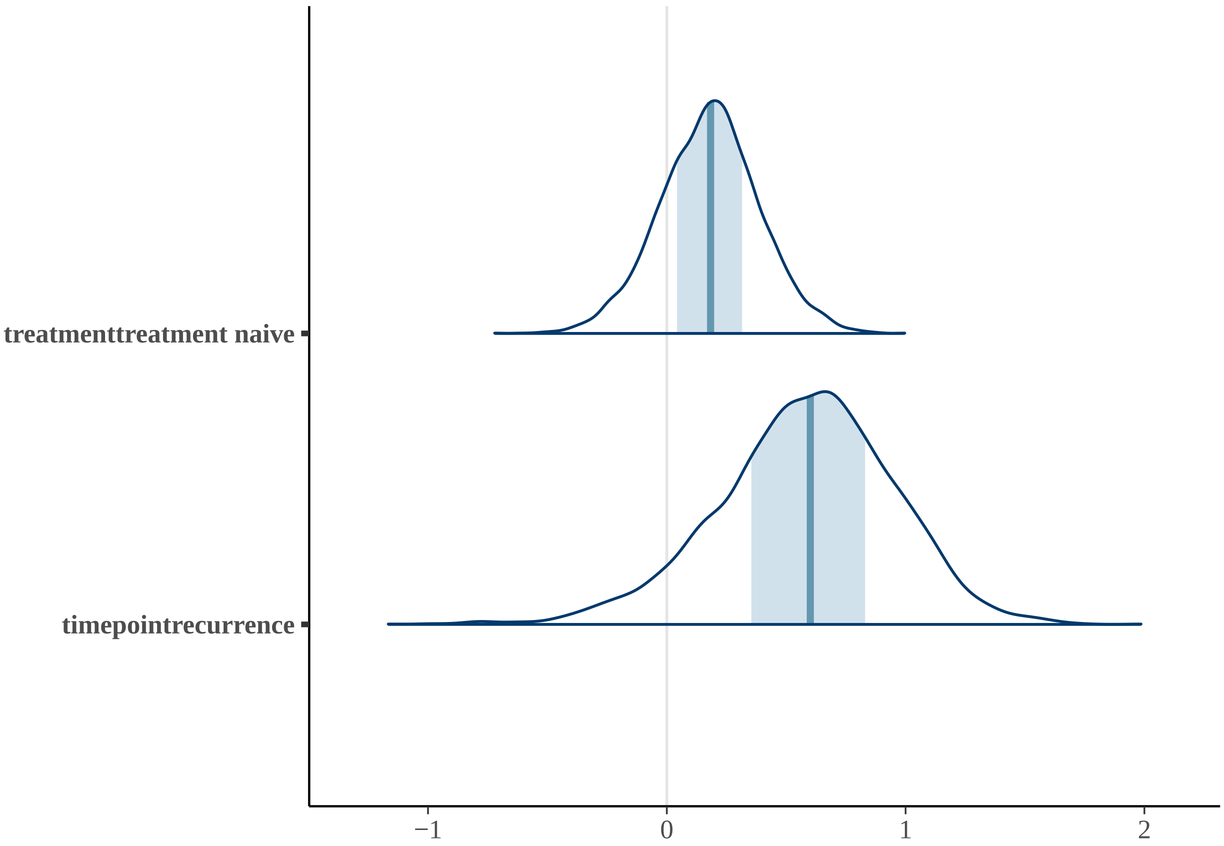

It is not surprising that these effects are similar to those estimated by the previous model, since most of our donors have only 1 sample. But it is reassuring to know that this particular modeling choice has little impact on our findings.

Here, giving a textual summary again

```
rstanarm::posterior_interval(strt3, prob = 0.95, pars = c('timepointrecurrence'))
```

```
##                           2.5%    97.5%
## timepointrecurrence -0.2124145 1.256275
```

And, calculating the percentage of posterior density that is <= 0.

```
mean(sapply(as.array(strt3)[,,'timepointrecurrence'], FUN = function(x) x <= 0))
```

```
## [1] 0.06625
```

Finally, we can plot our posterior-predicted intervals for the three models thus far, overlaying them with the observed data.

This is a somewhat useful way to gut-check the model parameters.

```
# calculating the posterior-predicted values
strt3.ppred <- rstanarm::predictive_interval(strt3) %>%
  tbl_df(.)
strt3.median <- rstanarm::predictive_interval(strt3, 0.01) %>%
```

```r
  tbl_df(.) %>%
  dplyr::mutate(median = (`49.5%` + `50.5%`)/2) %>%
  dplyr::select(median)

md_solid3 <-
  md_solid %>%
  dplyr::bind_cols(strt3.ppred) %>%
  dplyr::bind_cols(strt3.median)

strt2.ppred <- rstanarm::predictive_interval(strt2) %>%
  tbl_df(.)
strt2.median <- rstanarm::predictive_interval(strt2, 0.01) %>%
  tbl_df(.) %>%
  dplyr::mutate(median = (`49.5%` + `50.5%`)/2) %>%
  dplyr::select(median)

md_solid2 <-
  md_solid %>%
  dplyr::bind_cols(strt2.ppred) %>%
  dplyr::bind_cols(strt2.median)
```

```r
## plotting posterior-predicted values, with observed datapoints
ggplot(md_solid3, aes(x = specific_treatment, y = log1p(mutations))) +
  geom_jitter() +
  geom_errorbar(aes(x = specific_treatment, ymin = `5%`, ymax = `95%`, colour = 'model: primary only'),
              data = md_primary_solid2 %>% dplyr::distinct(specific_treatment, .keep_all=T),
              alpha = 0.5) +
  geom_errorbar(aes(x = specific_treatment, ymin = `5%`, ymax = `95%`, colour = 'model: varying-slope')
              data = md_solid3 %>% dplyr::distinct(specific_treatment, .keep_all=T),
              alpha = 0.5) +
  geom_errorbar(aes(x = specific_treatment, ymin = `5%`, ymax = `95%`, colour = 'model: varying-int'),
              data = md_solid2 %>% dplyr::distinct(specific_treatment, .keep_all=T),
              alpha = 0.5) +
  theme_minimal()
```

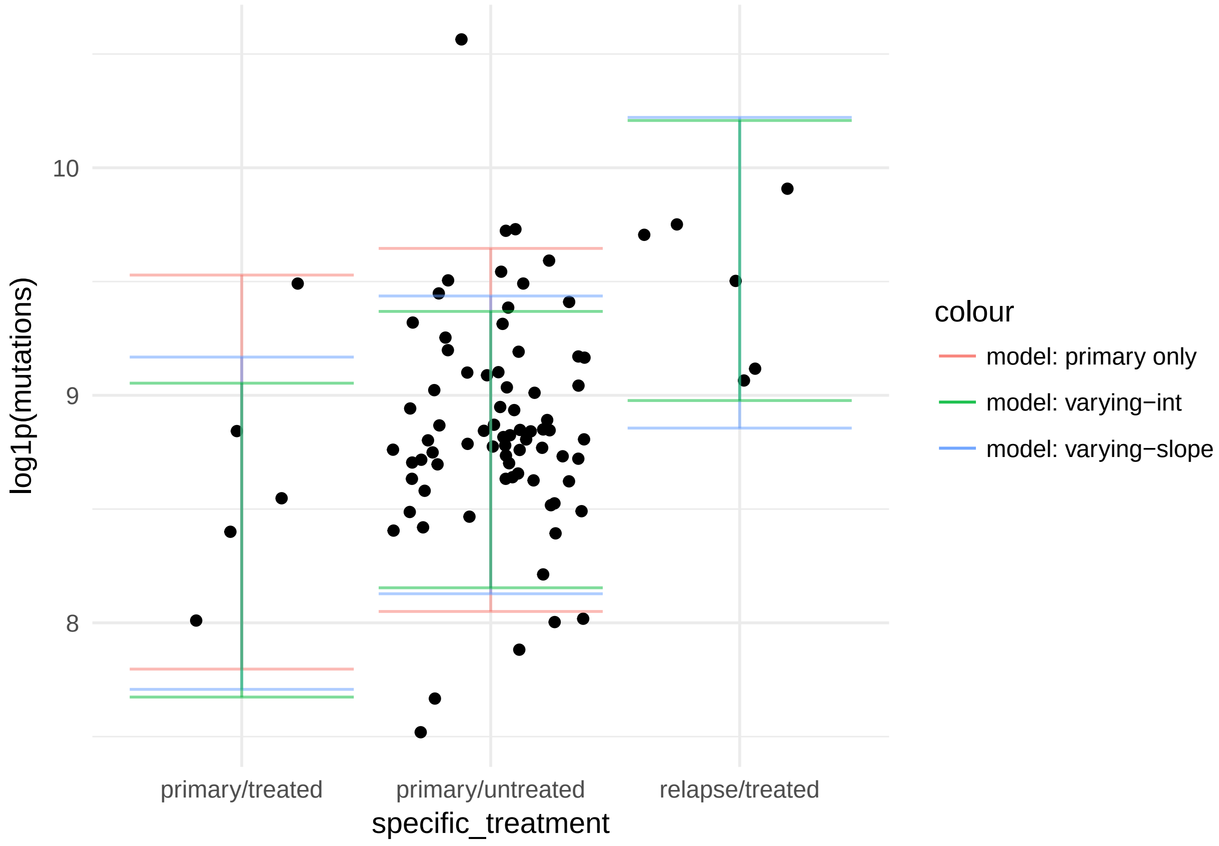

1.4 Including ascites samples

Finally we look at a model including ascites samples

```
atrt1 <- rstanarm::stan_glmer(log1p(mutations) ~
                                timepoint + treatment + (1 | donor) +
                                (1 + timepoint + treatment | tissue_type),
                              data = md,
                              seed = stan_seed,
                              adapt_delta = 0.999,
                              iter = 4000
                              )
```

```
## Warning: There were 5 divergent transitions after warmup. Increasing adapt_delta above 0.999 may hel
## http://mc-stan.org/misc/warnings.html#divergent-transitions-after-warmup
```

```
## Warning: There were 4 chains where the estimated Bayesian Fraction of Missing Information was low. S
## http://mc-stan.org/misc/warnings.html#bfmi-low
```

```
## Warning: Examine the pairs() plot to diagnose sampling problems
```

```
atrt1
```

```
## stan_glmer
```

```
##  family:  gaussian [identity]
##  formula: log1p(mutations) ~ timepoint + treatment + (1 | donor) + (1 +
##     timepoint + treatment | tissue_type)
## ------
##
## Estimates:
##                          Median MAD_SD
## (Intercept)              8.7    0.2
## timepointrecurrence      0.6    0.2
## treatmenttreatment naive 0.2    0.2
## sigma                    0.2    0.0
##
## Error terms:
##  Groups      Name                     Std.Dev. Corr
##  donor       (Intercept)              0.40
##  tissue_type (Intercept)              0.15
##              timepointrecurrence      0.14     -0.03
##              treatmenttreatment naive 0.14     -0.03  0.08
##  Residual                             0.20
## Num. levels: donor 92, tissue_type 2
##
## Sample avg. posterior predictive
## distribution of y (X = xbar):
##          Median MAD_SD
## mean_PPD 9.0    0.0
##
## ------
## For info on the priors used see help('prior_summary.stanreg').
```

It's worth noting that this model recovers the distribution of our original data pretty well.

```
bayesplot::pp_check(atrt1)
```

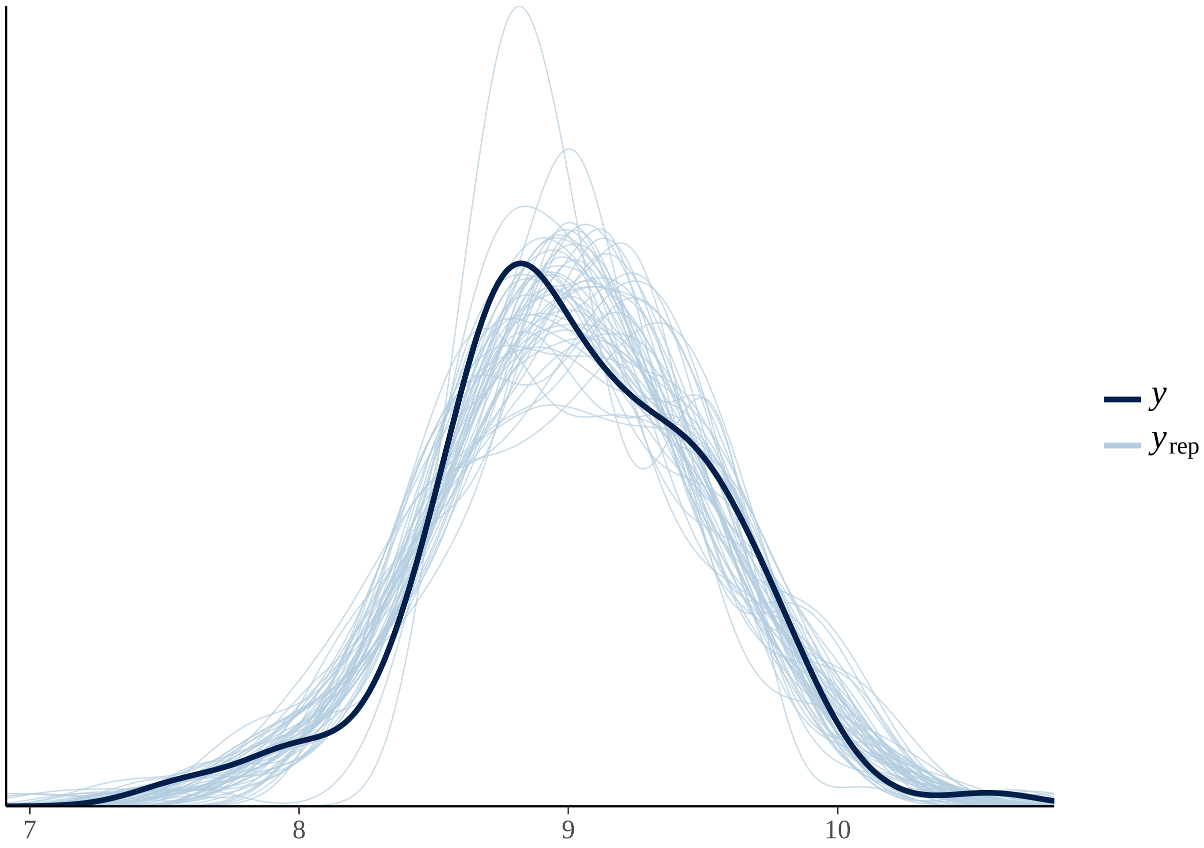

And, samples efficiently

```
bayesplot::mcmc_trace(as.array(atrt1), regex_pars = c('^treatment', '^timepoint'), facet_args = list(nc
```

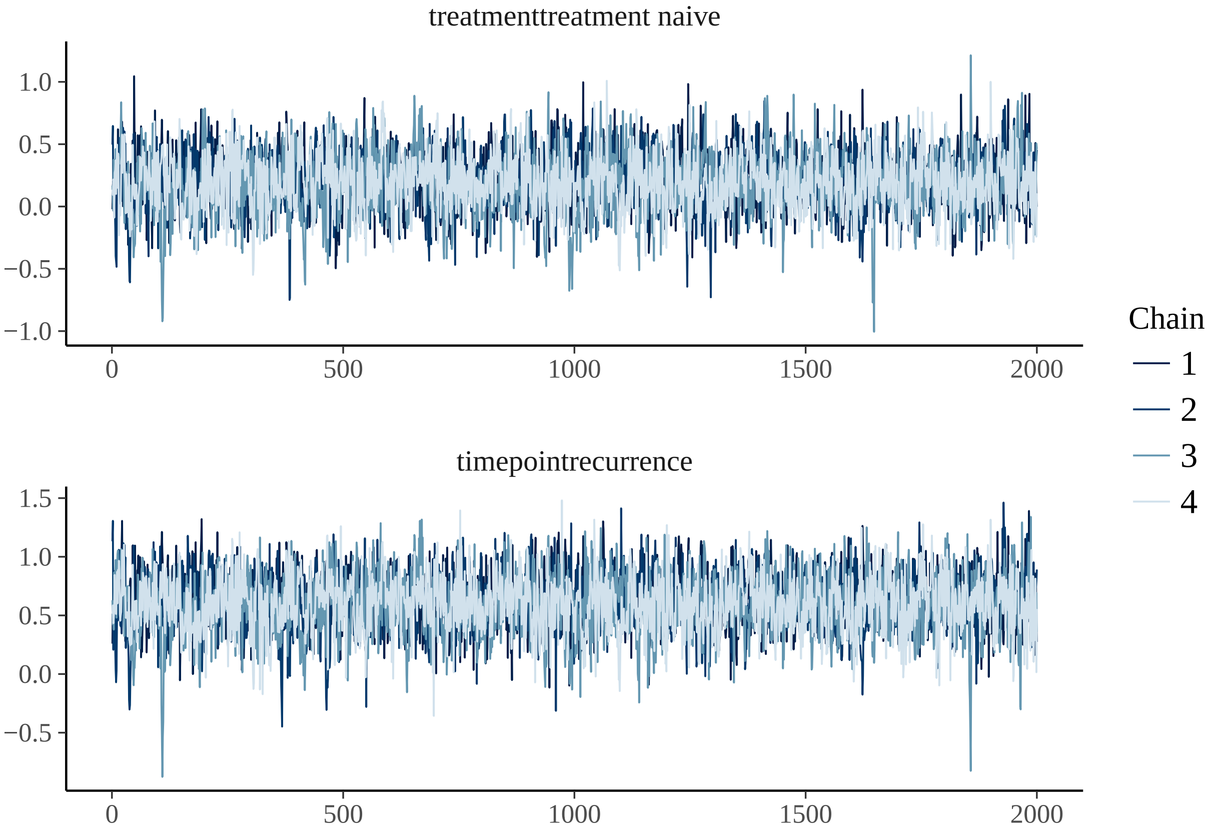

This model includes an overall estimate for treatment & timepoint effects, from which tissue-type-specific coeffients are drawn.

Here are the overall estimated effects.

```
bayesplot::mcmc_areas(as.array(atrt1), regex_pars = c('^treatment', '^timepoint'))
```

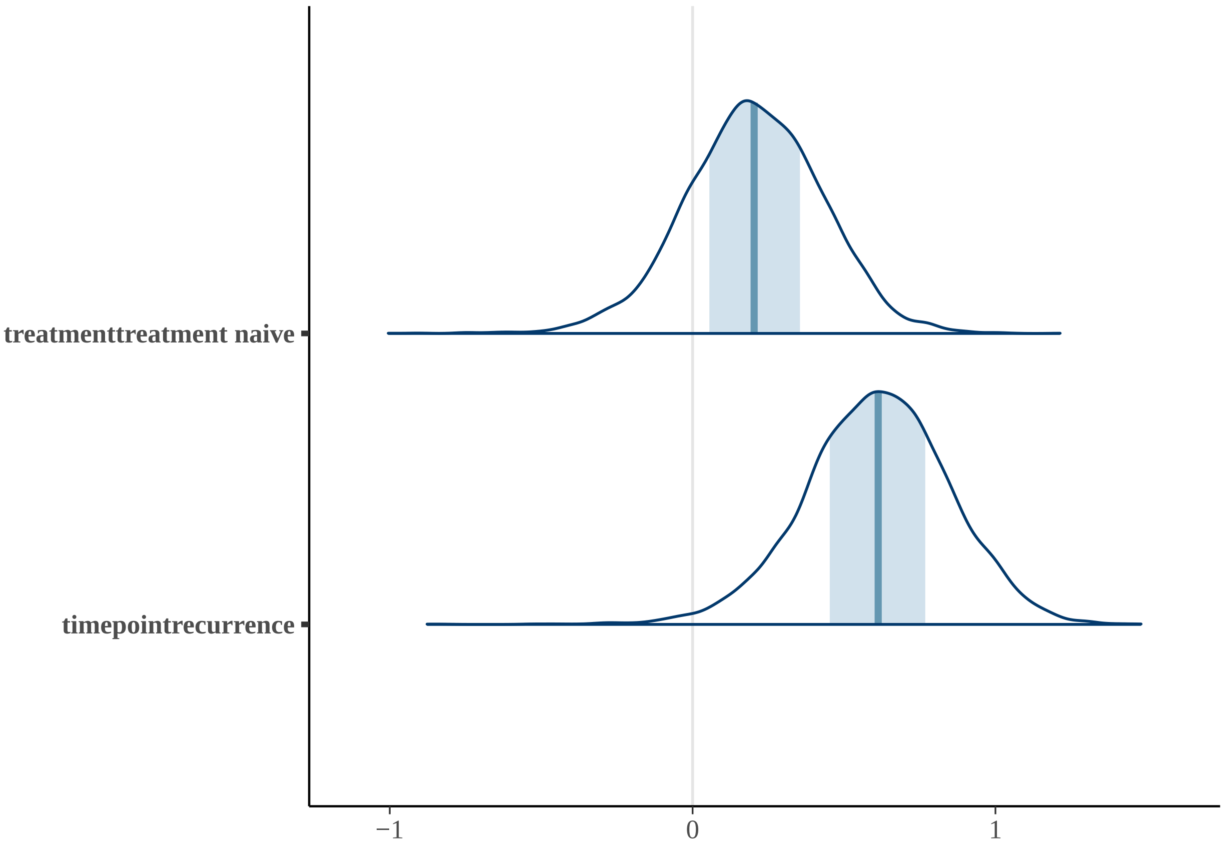

Again, they are directionally similar to the effects estimated in our subset analyses.

Let's compare the numerical summaries:

```
rstanarm::posterior_interval(atrt1, prob = 0.95, regex_pars = c('^timepoint', '^treatment'))
```

```
##                                2.5%    97.5%
## timepointrecurrence       0.1147481 1.062608
## treatmenttreatment naive -0.2621648 0.631993
```

The estimate for recurrent samples is higher in this model than was estimated among solid samples only.

```
rstanarm::posterior_interval(strt2, prob = 0.95, regex_pars = c('^timepoint', '^treatment'))
```

```
##                                 2.5%     97.5%
## timepointrecurrence      -0.05066051 1.1258756
## treatmenttreatment naive -0.25801920 0.5895171
```

How much of this probability mass is < 0?

```
mean(sapply(as.array(atrt1)[,,'timepointrecurrence'], FUN = function(x) x <= 0))
```

```
## [1] 0.010625
```

Let's look at the estimates per tissue type.

```r
bayesplot::mcmc_areas(as.array(atrt1), regex_pars = c('^b\\[timepoint'))
```

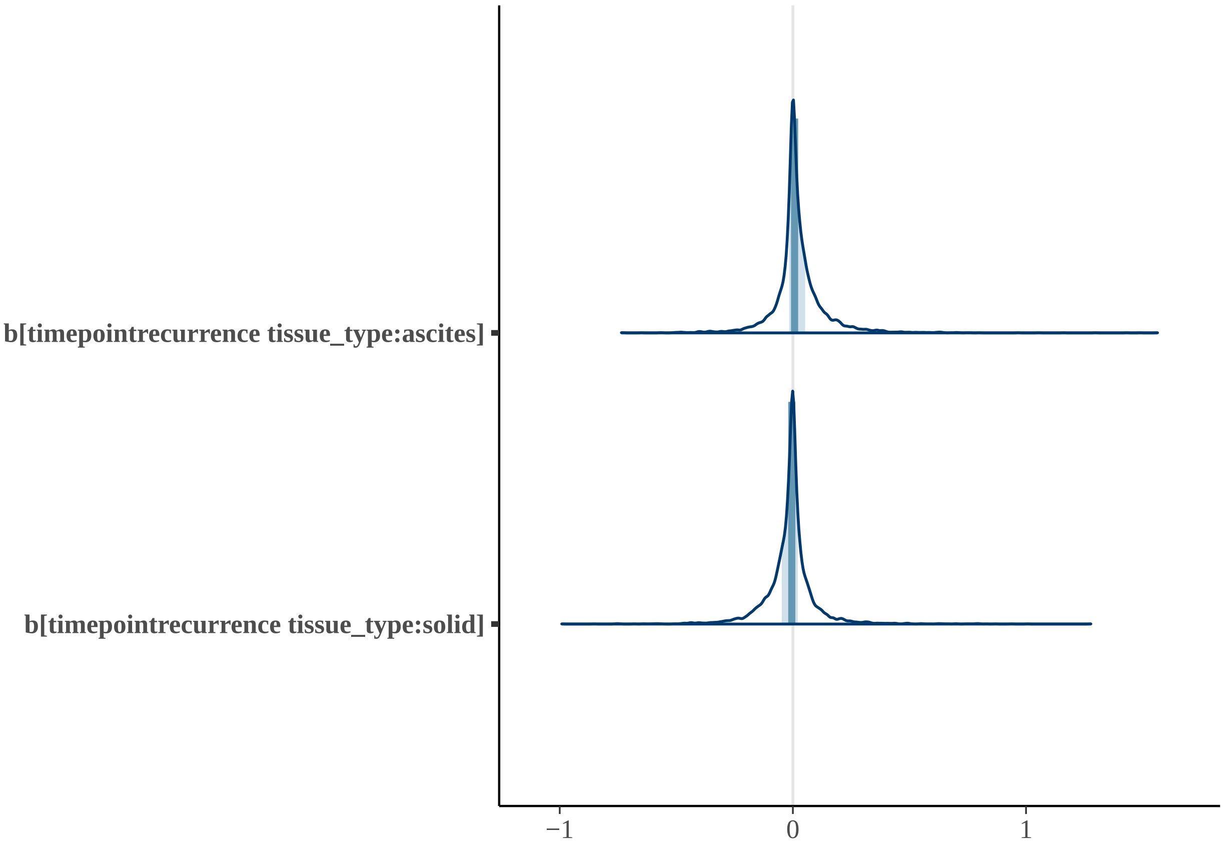

Interesting that, in this model, there is not a lot of variance of effects by tissue type.

Same goes for the treatment effect.

```r
bayesplot::mcmc_areas(as.array(atrt1), regex_pars = c('^b\\[treatment'))
```

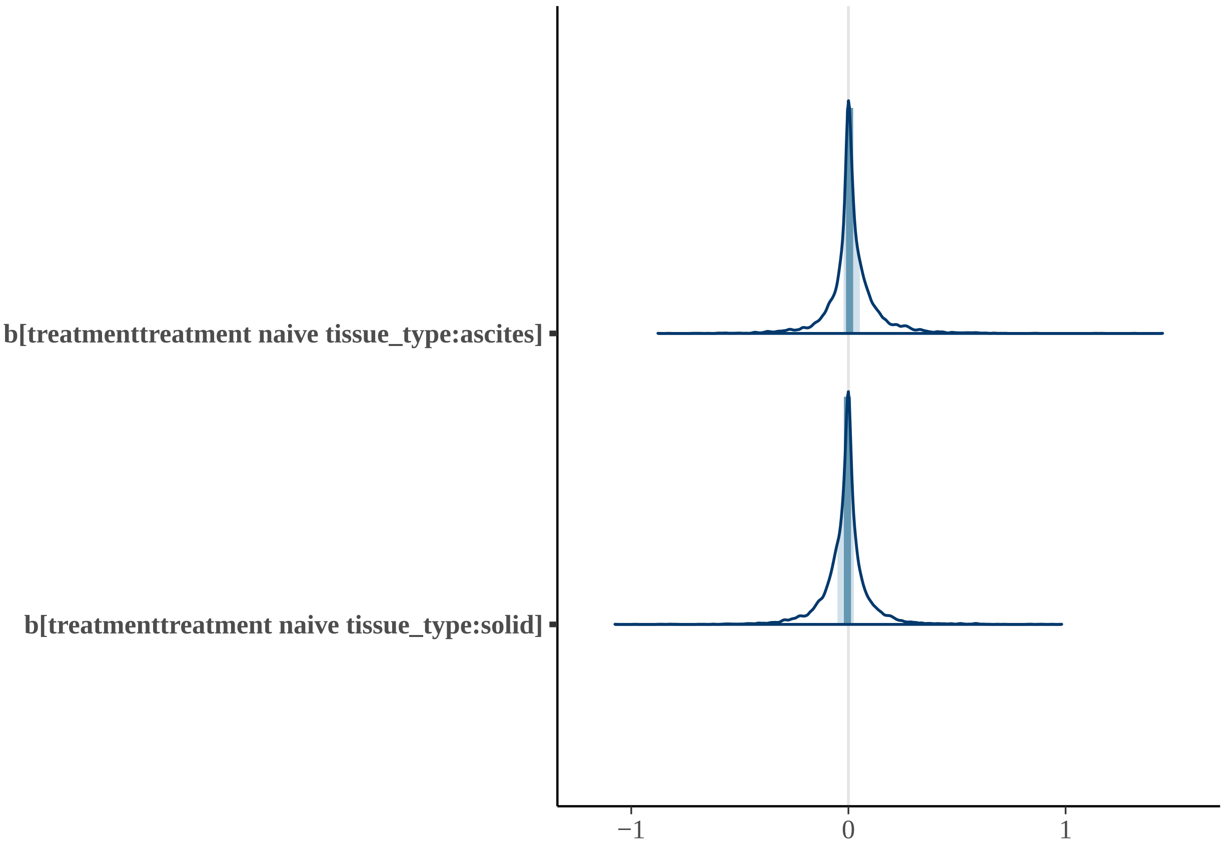

This could be due to a lack of sufficient data by which to estimate the variance, thus causing the model to fall back on default weakly informative priors which assume little variance by tissue type.

Let's now summarize our posterior-predicted values as we did in earlier models.

```
atrt1.ppred <- rstanarm::predictive_interval(atrt1) %>%
  tbl_df(.)
atrt1.median <- rstanarm::predictive_interval(atrt1, 0.01) %>%
  tbl_df(.) %>%
  dplyr::mutate(median = (`49.5%` + `50.5%`)/2) %>%
  dplyr::select(median)

md1 <-
  md %>%
  dplyr::bind_cols(atrt1.ppred) %>%
  dplyr::bind_cols(atrt1.median)
```

```
## plotting posterior-predicted values, with observed datapoints
ggplot(md1, aes(x = specific_treatment, y = log1p(mutations))) +
  geom_jitter() +
  facet_wrap(~tissue_type) +
  geom_errorbar(aes(x = specific_treatment, ymin = `5%`, ymax = `95%`, colour = 'model: primary only'),
               data = md_primary_solid2 %>% dplyr::distinct(specific_treatment, .keep_all=T),
               alpha = 0.5) +
  geom_errorbar(aes(x = specific_treatment, ymin = `5%`, ymax = `95%`, colour = 'model: primary solid')
               data = md_solid2 %>% dplyr::distinct(specific_treatment, .keep_all=T),
               alpha = 0.5) +
```

```
  geom_errorbar(aes(x = specific_treatment, ymin = `5%`, ymax = `95%`, colour = 'model: all samples'),
                data = md1 %>% dplyr::distinct(specific_treatment, tissue_type, .keep_all=T),
                alpha = 0.5) +
  theme_minimal()
```

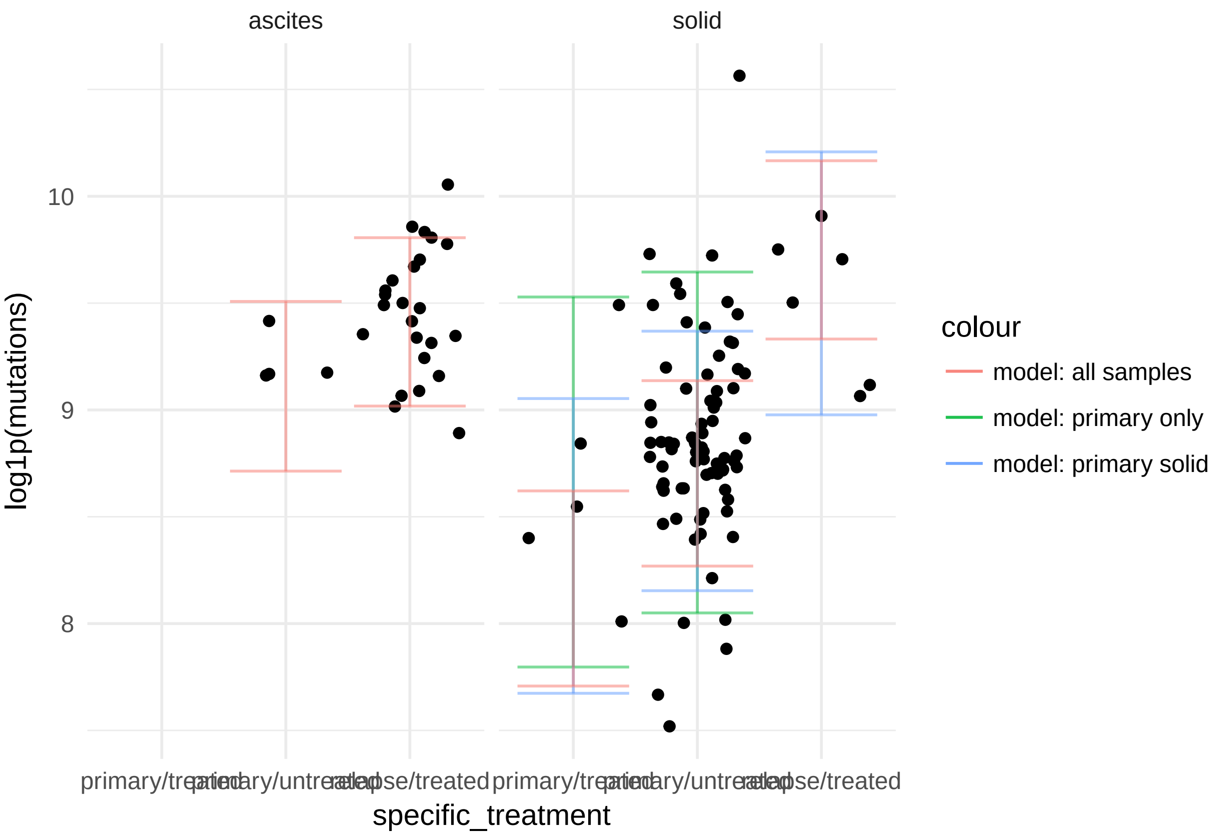

Strange how, with each additional sample type added to the model, the estimated mean primary/treated `log1p(mutation)` count gets lower.

1.4.1 Removing treatment from the model

What happens if we remove treatment from the model, and instead compare all relapse vs all primary (treated + untreated) samples?

```
atrt2 <- rstanarm::stan_glmer(log1p(mutations) ~
                                timepoint + (1 | donor) +
                                (1 + timepoint | tissue_type),
                              data = md,
                              seed = stan_seed,
                              adapt_delta = 0.999,
                              iter = 4000
                              )
```

```
## Warning: There were 1 divergent transitions after warmup. Increasing adapt_delta above 0.999 may hel
## http://mc-stan.org/misc/warnings.html#divergent-transitions-after-warmup

## Warning: There were 4 chains where the estimated Bayesian Fraction of Missing Information was low. S
## http://mc-stan.org/misc/warnings.html#bfmi-low
```

```
## Warning: Examine the pairs() plot to diagnose sampling problems
```

```
atrt2
```

```
## stan_glmer
##  family:  gaussian [identity]
##  formula: log1p(mutations) ~ timepoint + (1 | donor) + (1 + timepoint |
##     tissue_type)
## ------
##
## Estimates:
##                      Median MAD_SD
## (Intercept)          8.9    0.1
## timepointrecurrence  0.4    0.1
## sigma                0.2    0.0
##
## Error terms:
##  Groups      Name                Std.Dev. Corr
##  donor       (Intercept)         0.40
##  tissue_type (Intercept)         0.18
##              timepointrecurrence 0.17     0.03
##  Residual                        0.20
## Num. levels: donor 92, tissue_type 2
##
## Sample avg. posterior predictive
## distribution of y (X = xbar):
##          Median MAD_SD
## mean_PPD 9.0    0.0
##
## ------
## For info on the priors used see help('prior_summary.stanreg').
```

```
atrt2.ppred <- rstanarm::predictive_interval(atrt2) %>%
  tbl_df(.)
atrt2.median <- rstanarm::predictive_interval(atrt2, 0.01) %>%
  tbl_df(.) %>%
  dplyr::mutate(median = (`49.5%` + `50.5%`)/2) %>%
  dplyr::select(median)

md2 <-
  md %>%
  dplyr::bind_cols(atrt2.ppred) %>%
  dplyr::bind_cols(atrt2.median)
```

```
## plotting posterior-predicted values, with observed datapoints
ggplot(md2, aes(x = specific_treatment, y = log1p(mutations))) +
  geom_jitter() +
  facet_wrap(~tissue_type) +
  geom_errorbar(aes(x = specific_treatment, ymin = `5%`, ymax = `95%`, colour = 'model: all samples'),
              data = md1 %>% dplyr::distinct(specific_treatment, tissue_type, .keep_all=T),
              alpha = 0.5) +
  geom_errorbar(aes(x = specific_treatment, ymin = `5%`, ymax = `95%`, colour = 'model: recurrent only')
              data = md2 %>% dplyr::distinct(specific_treatment, tissue_type, .keep_all=T),
```

```r
                  alpha = 0.5) +
  theme_minimal()
```

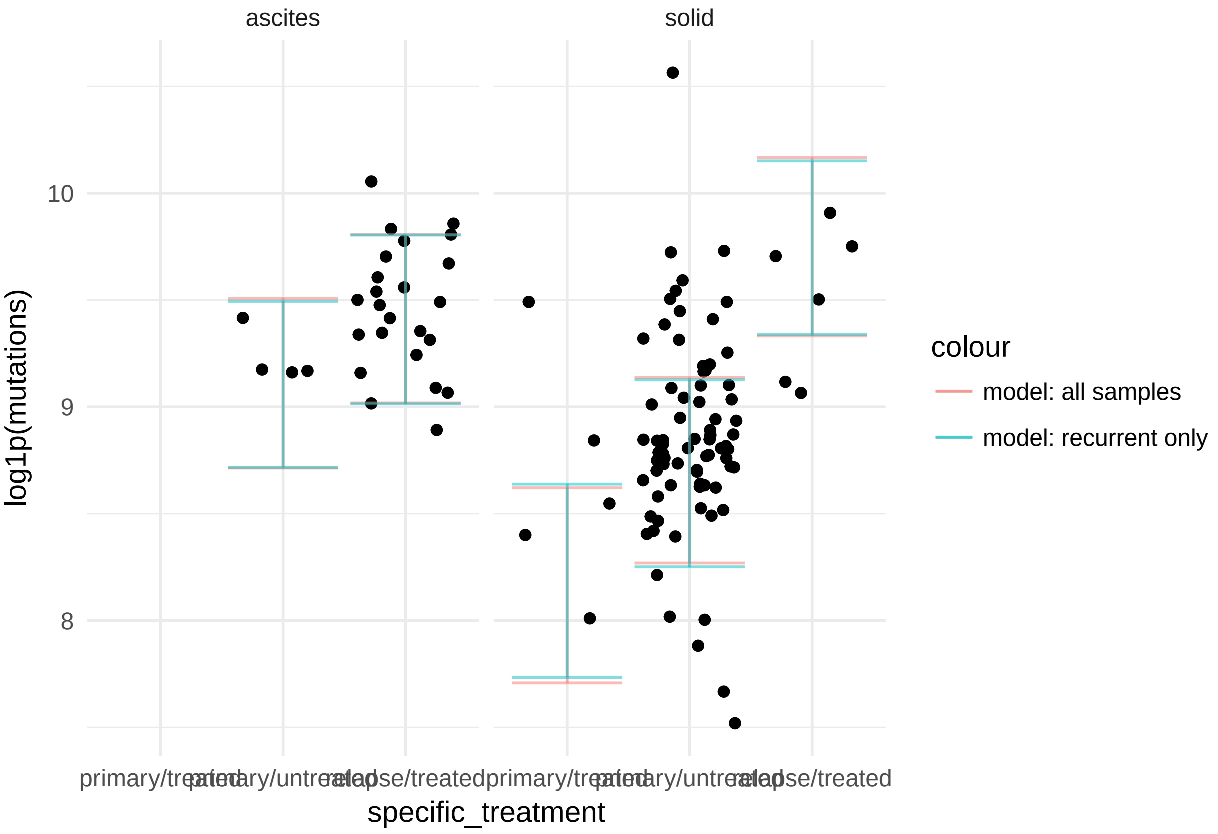

The only explanation for this must be that the scenarios with multiple samples per donor must have exaggerated the primary/treated vs primary/untreated effects in this model.

What is a numerical summary of this recurrence effect, in a univariate model adjusting only for multiple samples per donor & tissue type?

```r
rstanarm::posterior_interval(atrt2, prob = 0.95, regex_pars = c('^timepoint'))
```

```
##                            2.5%     97.5%
## timepointrecurrence 0.08891223 0.6694402
```

How much of this probability mass is < 0?

```r
mean(sapply(as.array(atrt2)[,,'timepointrecurrence'], FUN = function(x) x <= 0))
```

```
## [1] 0.01225
```

1.5 Analyzing peptides

1.5.1 Among solid samples

```r
f1 <- rstanarm::stan_glmer(log1p(peptides) ~
                              timepoint + treatment + (1 | donor),
                            data = md_solid,
                            seed = stan_seed,
                            adapt_delta = 0.999,
                            iter = 4000
                            )
```

```
## Warning: There were 4 chains where the estimated Bayesian Fraction of Missing Information was low. Se
## http://mc-stan.org/misc/warnings.html#bfmi-low

## Warning: Examine the pairs() plot to diagnose sampling problems
```

```r
f1
```

```
## stan_glmer
##  family:  gaussian [identity]
##  formula: log1p(peptides) ~ timepoint + treatment + (1 | donor)
## ------
##
## Estimates:
##                            Median MAD_SD
## (Intercept)                4.7    0.3
## timepointrecurrence        0.4    0.4
## treatmenttreatment naive 0.1    0.3
## sigma                      0.3    0.2
##
## Error terms:
##  Groups   Name        Std.Dev.
##  donor    (Intercept) 0.57
##  Residual             0.36
## Num. levels: donor 81
##
## Sample avg. posterior predictive
## distribution of y (X = xbar):
##          Median MAD_SD
## mean_PPD 4.9    0.0
##
## ------
## For info on the priors used see help('prior_summary.stanreg').
```

```r
mean(sapply(as.array(f1)[,,'timepointrecurrence'], FUN = function(x) x <= 0))
```

```
## [1] 0.131875
```

```r
rstanarm::posterior_interval(f1, prob = 0.95, regex_pars = c('^timepoint'))
```

```
##                             2.5%   97.5%
## timepointrecurrence -0.3180513 1.37191
```

1.5.2 Among all samples

```
f2 <- rstanarm::stan_glmer(log1p(peptides) ~
                                timepoint + treatment + (1 | donor) +
                                (1 + timepoint | tissue_type),
                              data = md,
                              seed = stan_seed,
                              adapt_delta = 0.999,
                              iter = 4000
                              )
```

```
## Warning: There were 1 divergent transitions after warmup. Increasing adapt_delta above 0.999 may hel
## http://mc-stan.org/misc/warnings.html#divergent-transitions-after-warmup

## Warning: There were 4 chains where the estimated Bayesian Fraction of Missing Information was low. S
## http://mc-stan.org/misc/warnings.html#bfmi-low

## Warning: Examine the pairs() plot to diagnose sampling problems
```

```
f2
```

```
## stan_glmer
##  family:  gaussian [identity]
##  formula: log1p(peptides) ~ timepoint + treatment + (1 | donor) + (1 +
##     timepoint | tissue_type)
## ------
##
## Estimates:
##                          Median MAD_SD
## (Intercept)              4.7    0.3
## timepointrecurrence      0.5    0.3
## treatmenttreatment naive 0.1    0.3
## sigma                    0.2    0.0
##
## Error terms:
##  Groups      Name                Std.Dev. Corr
##  donor       (Intercept)         0.62
##  tissue_type (Intercept)         0.19
##              timepointrecurrence 0.19     -0.04
##  Residual                        0.24
## Num. levels: donor 92, tissue_type 2
##
## Sample avg. posterior predictive
## distribution of y (X = xbar):
##          Median MAD_SD
## mean_PPD 5.0    0.0
##
## ------
## For info on the priors used see help('prior_summary.stanreg').
```

```r
mean(sapply(as.array(f2)[,,'timepointrecurrence'], FUN = function(x) x <= 0))
```

```
## [1] 0.086125
```

```r
rstanarm::posterior_interval(f2, prob = 0.95, regex_pars = c('^timepoint'))
```

```
##                           2.5%    97.5%
## timepointrecurrence -0.2184951 1.098158
```

1.5.3 Plot predicted values

```r
f1.ppred <- rstanarm::predictive_interval(f1) %>%
  tbl_df(.)
f2.ppred <- rstanarm::predictive_interval(f2) %>%
  tbl_df(.)

f1.md <-
  md_solid %>%
  dplyr::bind_cols(f1.ppred)
f2.md <-
  md %>%
  dplyr::bind_cols(f2.ppred)
```

```r
## plotting posterior-predicted values, with observed datapoints
ggplot(md, aes(x = specific_treatment, y = log1p(peptides))) +
  geom_jitter() +
  facet_wrap(~tissue_type) +
  geom_errorbar(aes(x = specific_treatment, ymin = `5%`, ymax = `95%`, colour = 'model: solid samples'),
                data = f1.md %>% dplyr::distinct(specific_treatment, tissue_type, .keep_all=T),
                alpha = 0.5) +
  geom_errorbar(aes(x = specific_treatment, ymin = `5%`, ymax = `95%`, colour = 'model: all samples'),
                data = f2.md %>% dplyr::distinct(specific_treatment, tissue_type, .keep_all=T),
                alpha = 0.5) +
  theme_minimal()
```

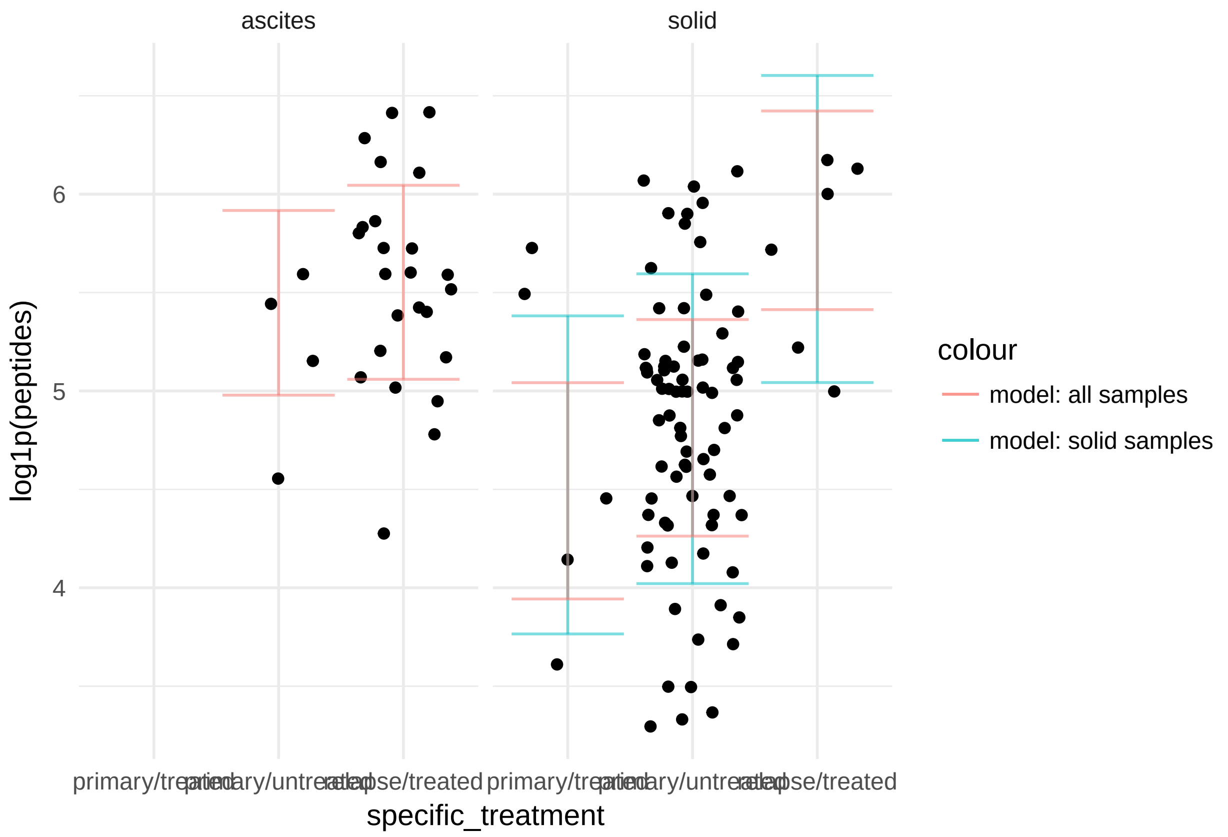
ascites
solid
log1p(peptides)
6
5
4
primary/treated
primary/untreated
relapse/treated
specific_treatment
colour
model: all samples
model: solid samples
